# High p62 expression suppresses the NLRP1 inflammasome and increases stress resistance in cutaneous SCC cells

**DOI:** 10.1038/s41419-022-05530-0

**Published:** 2022-12-29

**Authors:** Paulina Hennig, Michela Di Filippo, Gilles Bilfeld, Mark Mellett, Hans-Dietmar Beer

**Affiliations:** 1 Department of Dermatology, University Hospital Zurich, CH-8952 Schlieren, Switzerland; 2 Faculty of Medicine, University of Zurich, CH-8032 Zurich, Switzerland

**Keywords:** Squamous cell carcinoma, Inflammasome

## Abstract

NLRP1 is the primary inflammasome sensor in human keratinocytes. Sensing of UVB radiation by NLRP1 is believed to underlie the induction of sunburn. Although constitutive NLRP1 activation causes skin inflammation and predisposes patients to the development of cutaneous SCCs, the NLRP1 pathway is suppressed in established SCCs. Here, we identified high levels of the autophagy receptor p62 in SCC cells lines and SCC tumors. Increased NF-κB activity in SCC cells causes p62 up-regulation. Suppression of p62 expression rescues UVB-induced NLRP1 inflammasome activation in early-stage SCC cells. p62 expression protects SCC cells from cytotoxic drugs, whereas NLRP1 sensitizes them. In summary, we identify p62 as a novel negative regulator of the NLRP1 inflammasome in human cutaneous SCC cells, in which suppression of NLRP1 by increased levels of p62 supports stress resistance of skin cancer cells.

## Introduction

The skin represents the first line of defense against external insults [1]. Its outermost layer is the epidermis, a constantly renewing stratified epithelium consisting almost exclusively of keratinocytes. Keratinocyte-derived cancer is the most prevalent type of cancer worldwide comprising of basal and squamous cell carcinoma (BCC and SCC). Chronic skin inflammation and particularly high exposure to UVB radiation, which is completely absorbed by the epidermis, represent major risk factors for the development of SCCs [2]. On the other hand, immunosuppression predisposes patients to development of SCCs demonstrating an important role for anti-cancer immunity in keeping formation of SCCs under control [3].

UVB radiation is a complete carcinogen capable of inducing and promoting skin cancer. At the molecular level, DNA and RNA of epidermal keratinocytes are major chromophores for UVB radiation [4]. UV causes different types of photolesions either directly or upon generation of ROS. This activates a highly conserved network of pathways, termed the DNA damage response (DDR) [5]. Although very efficient, the DDR cannot repair all the UV-induced modifications correctly. Consequently, UV radiation leaves its marks in the DNA-causing mutations and, when these accumulate in basal keratinocyte stem cells, eventually skin cancer. In addition, UVB radiation has profound immunosuppressive effects, which are believed to contribute to skin cancer development [4].

UVB activates the NLRP1 (nucleotide-binding domain, leucine-rich-containing family, pyrin domain-containing-1) inflammasome upon phosphorylation of NLRP1 in human keratinocytes, which is believed to underlie the onset of sunburn [6, 7]. Interestingly, this is linked to absorption of UVB by RNA [6, 7]. Inflammasomes comprise a group of protein complexes, which assemble upon detection of certain stress factors by sensor proteins, such as NLRP1, NLRP3 or AIM2 (absent in melanoma 2) [8]. Upon oligomerization of the adaptor protein ASC (apoptosis-associated speck-like protein containing a caspase activation domain), the protease caspase-1 is activated. In turn, caspase-1 cleaves and activates the pro-inflammatory cytokines proIL-1β and -18 which release induces an inflammatory response. In addition, caspase-1 activates gasdermin D (GSDMD) causing its oligomerization and pore formation in the cell membrane required for the release of IL-1β and -18 that lack a signal peptide for conventional secretion by the endoplasmic reticulum-Golgi network. Furthermore, GSDMD induced pore formation can lead to a lytic and pro-inflammatory type of cell death termed pyroptosis. Inflammasomes are required for immunity but their activation also contributes to several common inflammatory diseases [9]. In contrast, the roles of inflammasomes in cancer development are incompletely understood [10].

Inflammasomes are expressed in a range of cells, including immune cells as well as epithelial cells, including keratinocytes. NLRP1 is the principal inflammasome sensor in human keratinocytes [11, 12]. In contrast, murine keratinocytes express neither major amounts of Nlrp1b, the most similar paralogue of NLRP1 in mice, nor proIL-1β demonstrating that the NLRP1 inflammasome in human keratinocytes represents a relatively young evolutionary pathway [13]. In addition, only human NLRP1 but not murine Nlrp1b is activated by viral 3 C proteases, dsRNA, and phosphorylation [6, 7, 14, 15]. Most importantly, patients with gain-of-function mutations of *NLRP1* suffer from inflammatory skin syndromes [11]. Furthermore, these mutations predispose patients to the development of SCCs suggesting that the inflammasome sensor acts as a tumor promoter [11]. However, the role of NLRP1 in skin cancer seems to be more complex because expression of the NLRP1 inflammasome and of proIL-1β is suppressed in human SCC cell lines and tumor biopsies [16].

The p62 protein, by its role as cargo receptor in autophagy, antagonizes the inflammasome pathway [17]. This occurs by autophagy-dependent degradation of ubiquitinated inflammasome proteins [18]. In addition, damaged mitochondria that induce NLRP3 activation are targeted by p62 for mitophagic degradation [19]. p62 itself is also degraded by autophagy and, therefore, its protein level might inversely correlate with autophagy flux. However, p62 changes can also occur independently of autophagy [20]. Due to its multi-domain structure, p62 regulates additional pro- and anti-inflammatory pathways, including the cytoprotective transcription factor Nrf2, the pro-inflammatory transcription factor NF-κB as well as the kinase mTOR that controls cell proliferation and differentiation via the mTORC1 complex [20, 21]. Most importantly, p62 expression is upregulated in different types of cancer cells, such as hepatocellular carcinoma (HCC), lung and colorectal cancer. In HCC, p62 supports carcinogenesis mainly through the activation of Nrf2 [22, 23] but also mTORC1 has been shown to play a role [24]. In papillary thyroid cancer, activation of mTOR by p62 contributes to tumor formation [25] and in lung adenocarcinomas high levels of p62 induce pro-tumorigenic NF-κB activation [26]. In human cutaneous SCCs, expression of p62 is suppressed in cancer-associated fibroblasts due to increased autophagy [27]. In contrast, SCC cells might express high p62 levels compared to healthy skin [28] but there is also evidence for p62 suppression upon tumor progression [29].

In this study, we identified high levels of p62 in human SCC cell lines and in SCC tumor biopsies. High p62 levels suppress NLRP1 and proIL-1β expression and are associated with increased stress resistance of SCC cells. This is partially mediated by NLRP1 suppression, whereas p62 regulation of the Nrf2, NF-κB, mTOR or autophagy pathways seems to be less relevant. In summary, in cutaneous SCCs we identified a pro-tumorigenic role for high p62 levels by increasing stress resistance which is partially mediated by suppression of the NLRP1 inflammasome pathway.

## Results

### p62 expression is induced in cutaneous SCCs

The NLRP1 pathway supports the development of SCCs, as patients with increased NLRP1 activity suffer not only from inflammatory skin syndromes but have an increased probability to develop SCC [11]. In contrast, expression of NLRP1 inflammasome components and proIL-1β is suppressed in human SCC cell lines and SCC tumors, in part by promoter methylation [16].

**Fig. 1:**
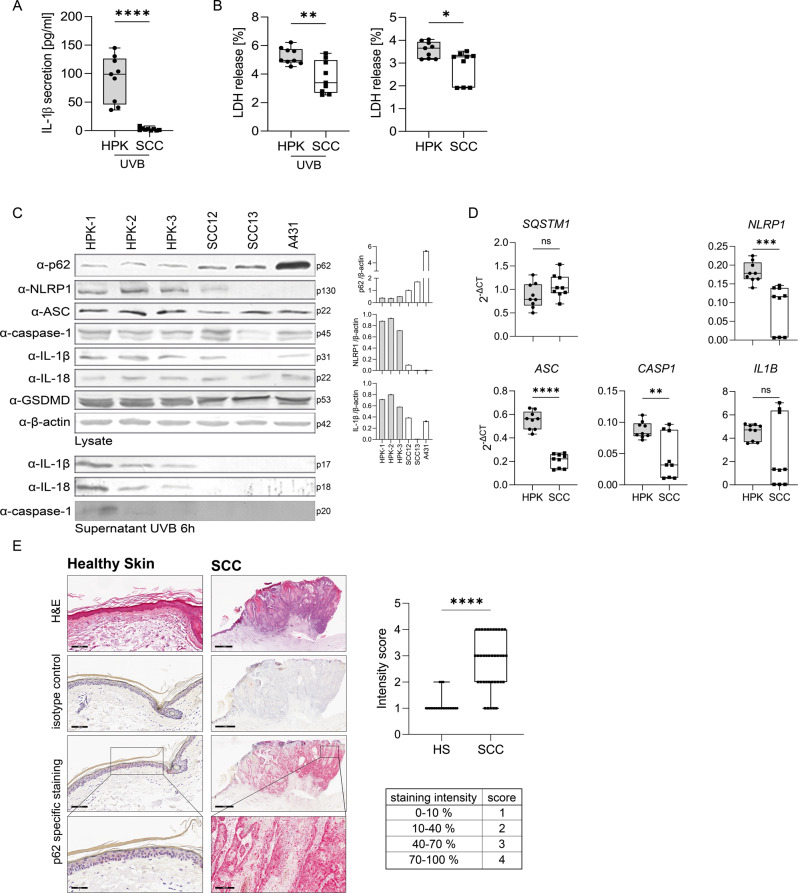
p62 expression is increased in SCC cells and carcinomas. **A**–**D** HPKs from three different donors were compared with SCC12, SCC13 and A431 cells. **A** IL-1β secretion determined by ELISA 6 h after UVB irradiation. **B** Cell lysis determined by LDH assay of UVB irradiated and mock-treated cells (after 6 h). **C** Western blots of lysate and of supernatant 6 h after UVB irradiation or mock treatment from HPKs and SCC cell lines (left panel) with a quantification of the band corresponding to p62, NLRP1, and IL1β normalized to β-actin (right panel). Representative experiment out of three. **D** mRNA expression of the indicated genes determined by qPCR in mock-treated cells. **E** H&E staining and p62 expression in healthy human skin (*N* = 22) and in SCCs (*N* = 38) isolated from patients determined by immunohistochemistry. A representative staining is shown. Expression of p62 was quantified and summarized by a blinded scoring system by two uninvolved individuals. Data are represented as mean ± SD of three experiments where 3 donors in the group of HPKs (*N* = 9) and 3 SCC cell lines (SCC12, SCC13, A431) in the group of SCCs (*N* = 9) were subjected (**A**, **B**, **D**) or are representative of three independent experiments (**C**). P values were calculated with two-tailed unpaired *t*-test (**A**, **B**, **D**) and Mann-Whitney test (**E**). (****P < 0.001, ***P ≤ 0.001, **P ≤ 0.01, and *P ≤ 0.05, ns = not significant). Black scale bar = 100 µm (healthy skin), 2 mm (SCC), magnitude 50 µm (healthy skin), 300 µm (SCC). SCC squamous cell carcinoma, HPK human primary keratinocyte, HS healthy skin.

Among SCC cell lines, SCC12 cells are most similar to human primary keratinocytes (HPKs) as both cell types grow in monoculture only in a low Ca^2+^-containing keratinocyte-specific medium. In contrast, SCC13 cells can be cultivated in either low Ca^2+^ keratinocyte medium or high Ca^2+^ DMEM, whereas A431 proliferate only in the latter [16]. Therefore, in the order SCC12, SCC13 and A431 the cells reflect increasing stages of cancer development. First, we confirmed that in HPKs UVB irradiation induces NLRP1 inflammasome activation, reflected by activation of caspase-1 and secretion of mature IL-1β and -18, using cells isolated from three different donors, but there was no secretion in the three SCC cell lines (Fig. 1A, C). Most likely, the failure to activate NLRP1 is caused by reduced expression of NLRP1 and ASC at the mRNA (Fig. 1D) and protein level (Fig. 1C) in all SCC cell lines. In addition, in SCC13 and A431 expression of caspase-1 and proIL-1β is reduced at the mRNA and the latter also at the protein level. p62 is a known antagonist of the inflammasome pathway and its expression is induced in different types of cancer [21]. Therefore, we wondered whether high p62 levels in SCC cells might contribute to suppression of the NLRP1 inflammasome. Indeed, particularly at the protein level, in the order SCC12, SCC13, and A431 expression of p62 is increased in SCC cell lines compared to HPKs (Fig. 1C, D). Most importantly, we identified significantly higher levels of p62 protein in patient-derived SCCs compared to healthy skin (Fig. 1E). In contrast, expression of p62 is not induced in BCCs (Supplementary Fig. S1).

With these experiments, we identified increased p62 expression in SCC cell lines (compared to HPKs) as well as in SCC tumors (compared to normal skin and BCCs).

### Suppression of p62 rescues UVB-induced NLRP1 inflammasome activation in SCC cells

**Fig. 2:**
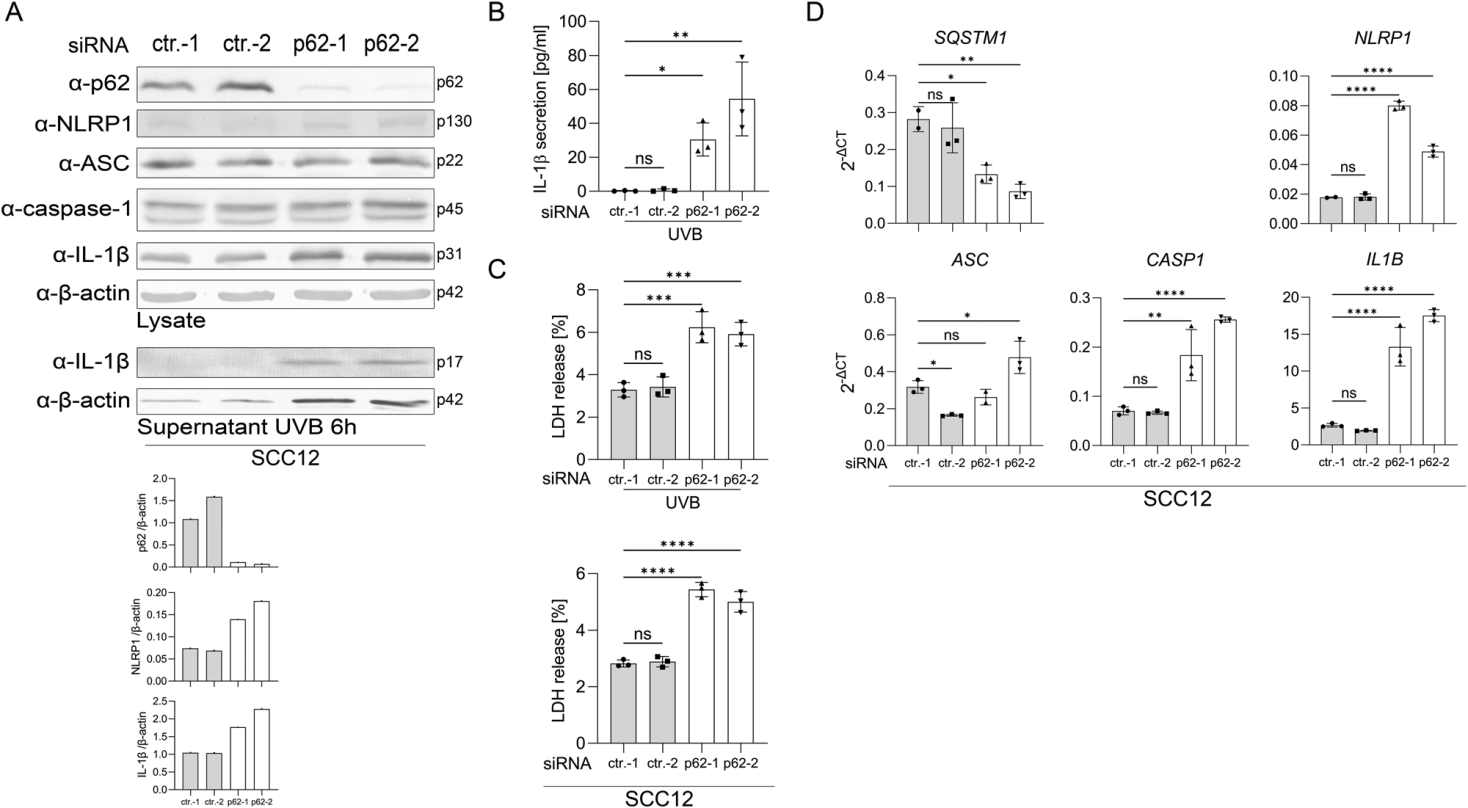
Knockdown of p62 expression rescues inflammasome activation in SCC12 cells. **A**–**C** SCC12 cells were transfected with two different control siRNAs (ctr.-1, ctr.-2) or two different siRNAs targeting p62 mRNA (p62-1, p62-2). **A** Western blots of lysate of mock-treated transfected SCC12 with a quantification of the band corresponding to p62, NLRP1, and IL1β normalized to β-actin (lower panel), and of supernatant 6 h after UVB irradiation. **B** IL-1β in the supernatant 6 h after UVB irradiation determined by ELISA. **C** LDH release 6 h after UVB irradiation (upper panel) or after mock-treatment. **D** mRNA expression of the indicated genes determined by qPCR in mock-treated cells. Data are represented as mean ± SD of three experiments using one-way analysis of variance with Dunnett’s multiple comparison test (*N* = 3) (**B**–**D**) or are representative of at least three independent experiments (**A**). (****P < 0.001, ***P ≤ 0.001, **P ≤ 0.01, and *P ≤ 0.05, ns = not significant).

Next, we speculated whether increased p62 expression in SCC cell lines dampens the NLRP1 pathway. Indeed, in the SCC12 cell line suppression of p62 expression by transfection of two different siRNAs rescued UVB-induced IL-1β secretion and, therefore, NLRP1 inflammasome activation (Fig. 2A, B). Most likely, this is caused by increased protein (Fig. 2A) and mRNA (Fig. 2D) expression of proIL-1β and NLRP1 upon p62 knockdown. Interestingly, the p62 knockdown has cytotoxic effects in SCC12 cells. The cells are characterized by a different morphology, a reduced growth rate (not shown), and more lysis, as they release more LDH to the medium (Fig. 2C). We observed a very similar phenotype in SCC12 cells upon knockout of p62 expression (Supplementary Fig. S2). In contrast, p62 knockdown or knockout in the SCC cell lines SCC13 and A431 increased NLRP1 and proIL-1β expression but could not rescue UVB-induced NLRP1 inflammasome activation and IL-1β secretion (not shown), demonstrating that additional mechanisms, such as promoter methylation, contribute to NLRP1 inflammasome suppression in these SCC cell lines [16].

In conclusion, high levels of p62 are responsible for suppression of the NLRP1 inflammasome pathway in the SCC12 cell line. p62 does not only prevent UVB-induced IL-1β secretion in these cells but has also a positive impact on cell viability.

### p62 is a negative regulator of the NLRP1 inflammasome in keratinocytes

**Fig. 3:**
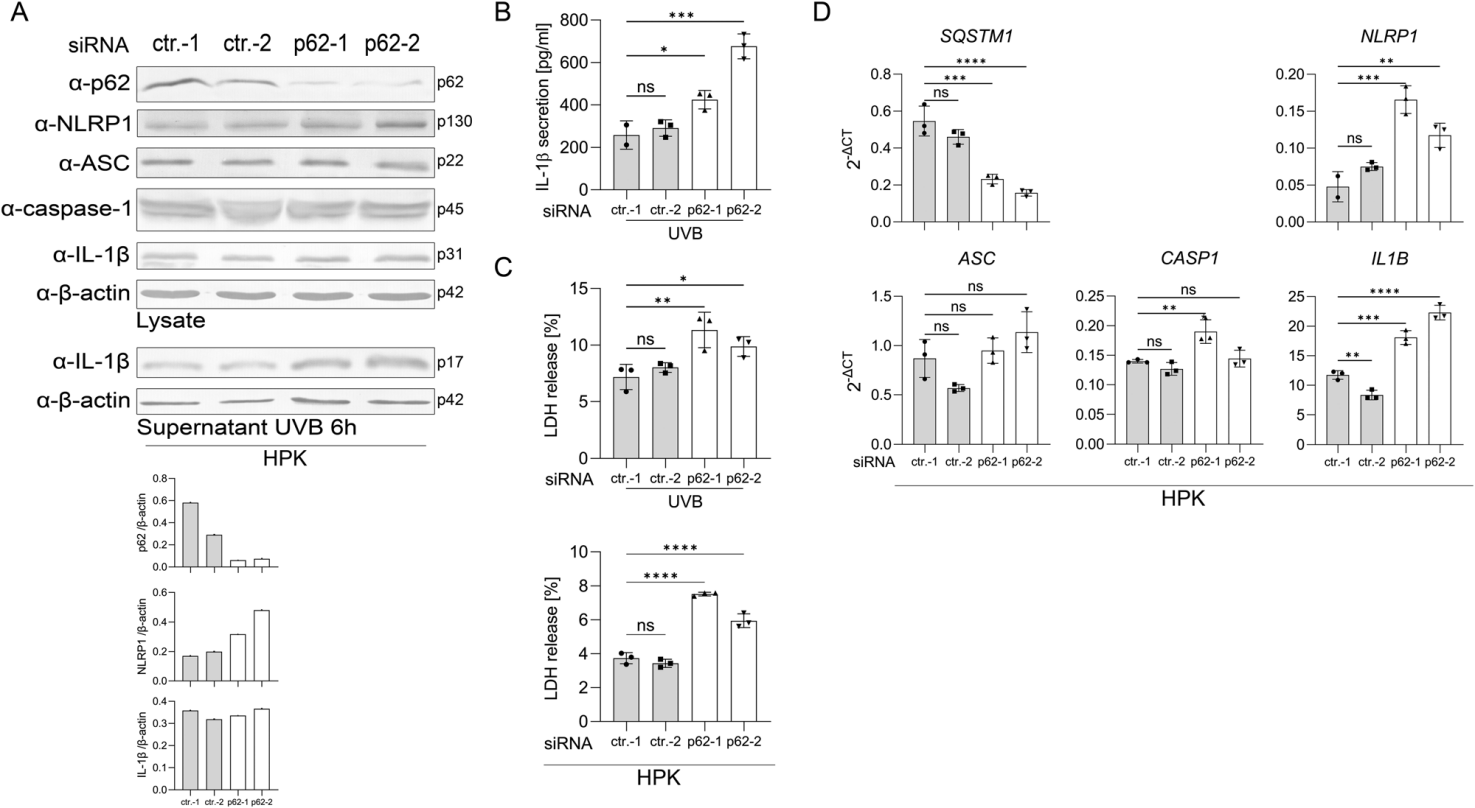
p62 knockdown supports inflammasome activation in HPKs. **A**–**D** HPKs were transfected with two control siRNAs (ctr.-1, ctr.-2) or two different siRNAs targeting p62 mRNA (p62-1, p62-2). **A** Western blots of lysates of mock-treated HPKs with a quantification of the band corresponding to p62, NLRP1, and IL1β normalized to β-actin (lower panel), and of supernatant 6 h after UVB irradiation. **B** ELISA measurement of IL-1β in the supernatant 6 h after UVB irradiation. **C** Cell lysis determined by LDH assay 6 h after UVB irradiation (upper panel) or upon mock-treatment (lower panel). **D** Expression of mRNA of the indicated genes in mock-treated cells determined by qPCR. Data are represented as mean ± SD of three experiments using one-way analysis of variance with Dunnett’s multiple comparison test (*N* = 3) (**B**–**D**) or are representative of at least three independent experiments (**A**). (****P < 0.001, ***P ≤ 0.001, **P ≤ 0.01, and *P ≤ 0.05, ns = not significant).

For characterization of the role of p62 in keratinocytes, we performed similar knockdown experiments with HPKs. UVB-irradiated p62 knockdown HPKs secreted more mature IL-1β (Fig. 3A and B), most likely due to increased expression of NLRP1 and proIL-1β (Fig. 3A and D). Also in these cells, suppression of p62 expression reduced the growth rate, altered cell morphology (not shown), and had cytotoxic effects reflected by more cell lysis (Fig. 3C). Moreover, overexpression of p62 in HPKs reduced IL-1β secretion induced by UVB irradiation (Supplementary Fig. S3). Therefore, in HPKs, levels of p62 are inversely correlated with the ability to activate the NLRP1 inflammasome.

### Autophagy, mTORC1, and Nrf2 are not significantly deregulated in SCC cells

p62 itself is degraded by autophagy and, therefore, its expression frequently reflects autophagic flux [30]. Inhibition of autophagy or overexpression of p62 in the liver is sufficient to induce liver cancer [31]. As p62 expression is strongly increased in SCC cell lines compared to HPKs (Fig. 1C) we queried whether this is a consequence of reduced autophagy. Therefore, we inhibited autophagy in HPKs and SCC cell lines by treatment with chloroquine and analyzed expression and localization of LC3B, a marker for autophagosomes [32, 33]. Chloroquine induced a similar accumulation of p62 and LC3B in HPKs and SCC cell lines suggesting comparable rates of autophagic flux in all cell types (Supplementary Fig. S4A and B).

mTORC1 is a downstream target of p62 and phosphorylates p70 S6 kinase at threonine 389 [34]. However, T389 of p70 S6 kinase is phosphorylated at similar levels in HPKs and SCC cell lines (Supplementary Fig. S4C) demonstrating that increased amounts of p62 in SCC cell lines do not induce mTORC1 activation.

**Fig. 4:**
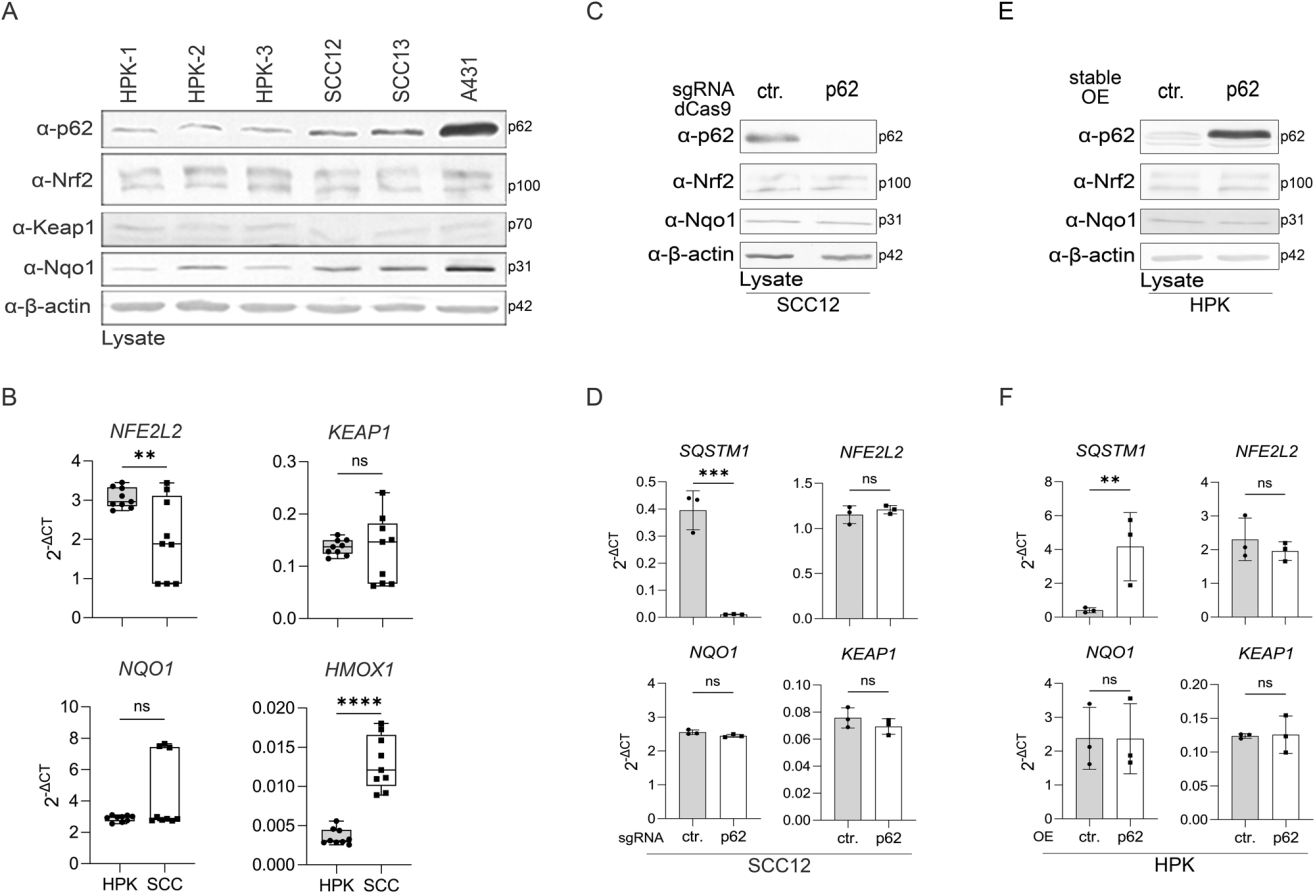
The Nrf2 pathway is not a target of p62 in SCC cells. HPKs from three different donors and SCC cell lines were analyzed for expression of the indicated genes. (**A**) Western blots for protein expression and (**B**) qPCR for mRNA expression. Expression of the indicated genes in control and p62 knockout SCC12 cells. **C** Western blots for protein and **D** qPCR for mRNA expression. **E**, **F** Expression of the indicated genes in control and stable p62 overexpressing HPKs. **E** Protein expression by western blots and **F** qPCR for mRNA expression. Data are represented as mean ± SD of three experiments (**B**, **D**, **F**) or are representative of three (**A**, **C**) or two (**E**) independent experiments. **B** 3 donors in the group of HPKs (*N* = 9) and 3 SCC cell lines (SCC12, SCC13, A431) in the group of SCCs (*N* = 9) were subjected. P values were calculated with two-tailed unpaired (**B**) or paired (*N* = 3) (**D**, **F**) *t* test. (****P < 0.001, ***P ≤ 0.001, **P ≤ 0.01, ns = not significant).

The cytoprotective transcription factor Nrf2 plays important roles in the skin [35]. Activation of Nrf2 is a frequent event in cancer and can be caused by somatic mutations in the *NRF2* gene as well as in the *KEAP1* gene [36]. In addition, increased p62 levels can activate Nrf2 by interaction and autophagic degradation of its inhibitor Keap1, as demonstrated in HCCs [22, 23]. Nrf2 is activated in different types of SCCs, in part by mutations of the DLG and ETGE motifs of Nrf2, which are responsible for interaction with Keap1, but the data concerning cutaneous SCCs are limited [37]. Sequencing of the *NRF2* and *KEAP1* genes in SCC cell lines revealed only silent mutations (Supplementary Fig. S4D). In order to analyze whether the Nrf2 pathway is a major downstream target of p62 in SCC cells we characterized SCC12 cells with knockout of p62 expression. However, p62 knockout did not affect expression and activity of Nrf2 (Fig. 4C and D). Similarly, overexpression of p62 in HPKs did not influence the Nrf2 pathway (Fig. 4E, F).

These experiments demonstrate that in HPKs and SCC cells autophagy, mTORC1 and Nrf2 are not major downstream or upstream pathways of p62. Additionally, upregulation of p62 expression in SCC cell lines is not a consequence of reduced autophagy in these cells.

### NF-κB induces p62 expression in SCC cell lines

**Fig. 5:**
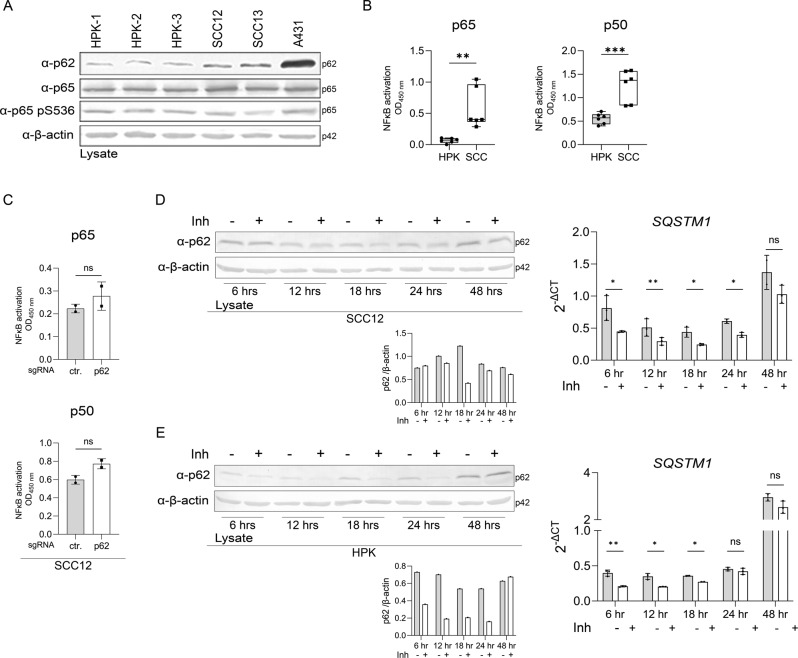
NF-κB activity is increased in SCC cells and regulates p62 expression. **A** Western blots for expression of the indicated genes in HPKs isolated from three different donors and in SCC cell lines SCC12, SCC13 and A431. **B** NF-κB activity assay for the indicated family members in HPKs and SCC cell lines. **C** p62 does not regulate NF-κB activity in SCC12 cells. NF-κB activity was determined in SCC12 cells lacking p62 expression and in control cells. SCC12 (**D**) and HPKs (**E**) were mock-treated or with the NF-κB inhibitor BMS-345541. Expression of p62 was analyzed at the protein level by western blot with a quantification of the band corresponding to p62 normalized to β-actin (lower panel), and at the mRNA level by qPCR. Data are represented as mean ± SD of two (**C**) or three (**B**, **D**, **E**) experiments, or are representative of three (**A**) or two (**D** left panel**, E** left panel) independent experiments. **B** 3 donors in the group of HPKs (*N* = 9) and 3 SCC cell lines (SCC12, SCC13, A431) in the group of SCCs (*N* = 9) were subjected. *P* values were calculated with two-tailed unpaired (**B**) or paired (**C** (*N* = 2)**, D** (*N* = 3)**, E** (*N* = 3)) *t*-test. (***P ≤ 0.001, **P ≤ 0.01, and *P ≤ 0.05, ns not significant).

The transcription factor NF-κB is linked to p62 in an upstream and downstream manner. On one hand, transcription of the *p62/SQSTM1* gene is regulated by NF-κB and on the other hand, p62 is an activator of NF-κB [20, 21]. Immunoblotting revealed that expression and activity of the NF-κB family member p65 is increased in SCC cell lines compared to HPKs (Fig. 5A). To address the roles of NF-κB in a quantitative manner we measured activity of NF-κB family members in HPKs and SCC cell lines. Interestingly, activity of p65 and p50 are increased in SCC cells compared to HPKs (Fig. 5B). Next, we addressed the question whether p62 levels, which are increased in SCC cell lines, contribute to enhanced NF-κB activity. However, in p62 knockout SCC12 cells (Supplementary Fig. S2) the activity of the NF-κB family members is not affected (Fig. 5C).

Transcription of the *p62/SQSTM1* gene is stress-associated and regulated by Nrf2, NF-κB and the MiT/TFE family of transcription factors [21]. In order to determine a possible contribution of NF-κB, to p62 protein levels in SCC cell lines, we inhibited its activity and analyzed p62 expression in SCC12 cells. Interestingly, in SCC12 cells and HPKs p62 protein and mRNA expression decreased upon inhibition of NF-κB (Fig. 5D), demonstrating that increased p62 levels in SCC cell lines are at least partially induced by the enhanced activity of NF-κB in these cells.

### p62 increases and NLRP1 reduces resistance against antineoplastic drugs

**Fig. 6:**
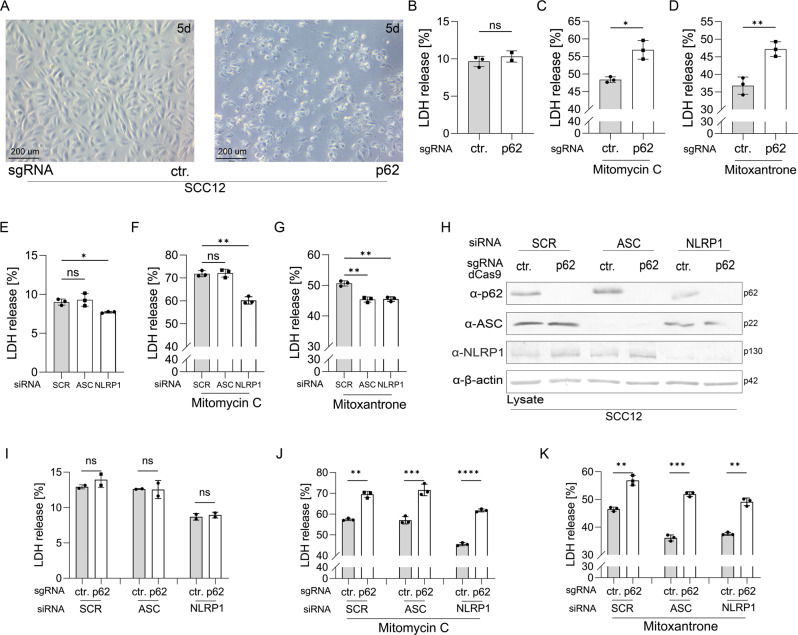
p62 protects and NLRP1/ASC sensitizes SCC12 cells against anti-neoplastic drugs. Control and p62 knockout SCC12 cells were mock-treated (**A**, **B**) or with mitomycin C or mitoxantrone for 24 h (**C**, **D**). **A** Microscopic picture of control (left panel) and p62 knockout SCC12 cells 5 days after seeding of same cell numbers. LDH release 24 h after **B** mock or **C** mitomycin C or **D** mitoxantrone treatment. SCC12 cells were transfected with scrambled siRNA (SCR) or siRNA targeting ASC or NLRP1 mRNA. LDH release after **E** mock or **F** mitomycin C or **G** mitoxantrone treatment. Control and p62 knockout SCC12 cells were transfected with scrambled, ASC, or NLRP1 mRNA targeting siRNA. **H** Western blot for expression of the indicated genes. LDH release 24 h after **I** mock-treatment or with **J** mitomycin C or **K** mitoxantrone. Data are represented as mean ± SD of three (**B**–**G**, **J**–**K**) or two (**I**) experiments, or are representative of three (**H**) independent experiments. P values were calculated with two-tailed paired (*N* = 3) (**B**–**D**, **I**–**K**) *t* test, or one-way analysis of variance with Dunnett’s multiple comparison test (*N* = 3) (**E**–**G**). (****P < 0.001, ***P ≤ 0.001, **P ≤ 0.01, and *P ≤ 0.05, ns not significant).

Next, we wondered whether SCC cells profit from high levels of p62. Knockout of p62 expression (Supplementary Fig. S2) clearly affected proliferation of mock-treated SCC12 cells in a negative manner (Fig. 6A), but cell death reflected by release of LDH was not changed (Fig. 6B). Treatment of SCC12 cells with the antineoplastic drugs mitomycin C or mitoxantrone induced cell death and release of LDH. Most importantly, knockout of p62 expression in these cells increased LDH release in a significant manner demonstrating that p62 confers protection against cytostatic drugs in SCC cells (Fig. 6C, D).

p62 is an autophagy receptor able to activate the mTORC1, Nrf2, and NF-κB pathway, which are all associated with carcinogenic conversion of epithelial cells. However, neither autophagy nor mTORC1 nor Nrf2 are significantly deregulated in SCC cells compared to HPKs (Supplementary Fig. S4 and Fig. 4) and NF-κB is activated independently of p62 (Fig. 5). To address whether the NLRP1 inflammasome contributes to cytoprotection we knocked down expression of ASC and NLRP1 in SCC12 cells. Interestingly, already mock-treated NLRP1 knockdown cells are characterized by lower levels of LDH release compared to control (transfected with scrambled siRNA) and ASC knockdown cells (Fig. 6E). Furthermore, a NLRP1 knockdown significantly protected mitomycin C-treated SCC12 cells whereas both NLRP1 and ASC suppression reduced LDH release after mitoxantrone treatment (Fig. 6G). In contrast, knockdown of NLRP1 or ASC expression in A431 cells, where expression of these proteins is lower compared to SCC12 cells (Fig. 1C, D) and inflammasome activation cannot be rescued, has no effect on cytoprotection (not shown).

Then, we tested whether p62-mediated cytoprotection and increased sensitivity of SCC12 cells against the antineoplastic drugs upon NLRP1/ASC knockdown are connected and knocked down expression of ASC and NLRP1 in p62 knockout SCC12 and control cells (Fig. 6H–K). p62 knockout increased cell death in mitomycin C and mitoxantrone-treated SCC12 cells in a similar manner irrespective of knockdown of ASC or NLRP1 expression, demonstrating that cytoprotection by p62 occurs independently of the NLRP1 pathway. In addition, a NLRP1 knockdown reduced cell death induced by mitomycin C or mitoxantrone treatment in control and p62 knockout cells. Knockdown of ASC expression has only an effect on mitoxantrone-induced cell death.

These experiments demonstrate that p62 expression supports survival of SCC12 cells treated with cytostatic drugs. Although high p62 expression in SCC12 cells reduces expression of NLRP1 and ASC, these reduced levels of the inflammasome proteins are sufficient to contribute to cell death.

## Discussion

p62 is a stress-induced, multi-functional, and multi-domain scaffold protein which expression is upregulated in different types of cancer [21, 38]. Due to its role as cargo receptor for auto-, mito-, and xenophagy, p62 levels are also regulated by degradation in autolysosomes. Accumulation of p62 protein in the initial phase of tumor development can result from a reduced autophagic flux. Autophagy is reported to be a tumor suppressor pathway in early tumor development, whereas in advanced stages it can promote survival of tumor cells [39]. In the liver, for example, different types of stressors inhibit autophagy, causing accumulation of p62 and, thereby, initiation of HCCs [40]. Upregulation of p62 can activate several pro-tumorigenic pathways, including the pro-inflammatory transcription factor NF-κB, the cytoprotective transcription factor Nrf2, and the kinase mTOR, a central regulator of proliferation, growth, metabolism, and survival [41]. Here, we demonstrate that in cutaneous SCC cells, NF-κB induces p62 expression that causes suppression of the NLRP1 inflammasome pathway.

Due to their key role in inflammation, inflammasomes are bona fide players in all stages of tumorigenesis [10]. For example, via caspase-1-dependent activation of proIL-1β and regulation of IL-1 secretion by GSDMD, inflammasomes support development of lung, gastric and mammary cancer, most likely through induction of inflammation and angiogenesis as well as suppression of anti-tumor immunity by IL-1β [42]. In contrast, caspase-1-activated IL-18 protects from colorectal cancer. Furthermore, inflammasome activation represents a death pathway, which, when activated in tumor cells, should antagonize tumor development [43]. Therefore, inflammasomes can support as well as inhibit tumor development depending on the cell type, which express and activate inflammasomes, the type of inflammasome complexes involved, and the stage of tumor development when inflammasome activation occurs. NLRP1 is the central inflammasome sensor in human keratinocytes and skin, and is activated upon UVB radiation-induced by NLRP1 phosphorylation [6, 7, 11, 44, 45]. NLRP1 is a tumor promoter, as patients with gain-of-function mutations of *NLRP1* suffer from inflammatory skin syndromes and have a predisposition for cutaneous SCCs [11]. Nevertheless, expression of NLRP1 inflammasome proteins and of proIL-1β is suppressed in cutaneous SCC cell lines and tumors, partially by promoter methylation [16]. Here, we demonstrate that upregulation of p62 expression in SCC cells in vitro and in carcinomas represents a second pathway that antagonizes the NLRP1 inflammasome, suggesting that NLRP1 suppression in SCCs is relevant for cancer development. Indeed, suppression of NLRP1 expression in SCC12 cells reduces lysis of mock- and with cytostatic drugs-treated cells, partially independent of ASC expression (Fig. 6E–G). This suggests that NLRP1 suppression might have inflammasome/ASC-dependent as well as -independent effects in SCC cells. Murine Nlrp1b can be activated independently of ASC expression causing IL-1β secretion [46]. Similarly, CARD8- and Nlrp1b-induced pyroptosis occurs independently on ASC expression [47, 48]. However, it is unlikely that NLRP1 and proIL-1β are suppressed by p62-dependent autophagy in SCCs, as suggested for other inflammasome proteins [17], since our experiments demonstrate that p62 regulates also mRNA expression of NLRP1 and proIL-1β.

In SCCs, p62 expression levels are increased in comparison to healthy human skin and to BCCs (Fig. 1E, Supplementary Fig. S1). This might be relevant for diagnostic purposes as well as for pharmacological therapy. Aldara is cream containing the Toll-like receptor 7 agonist imiquimod approved for actinic keratosis, BCCs, and precancerous SCCs (SCC in situ), but not recommended for the treatment of advanced SCCs [49]. Interestingly, Aldara activates the NLRP1 inflammasome in HPKs in an imiquimod-independent manner causing IL-1β secretion and cell death [50]. Upregulation of p62 expression in SCCs and its suppression of the NLRP1 pathway might represent the reason, why Aldara loses its therapeutic activity in patients suffering from advanced SCCs. Consequently, suppression of p62, e.g., by induction of autophagy, might rescue the therapeutic activity of Aldara in SCC patients.

SCC cells profit not only via suppression of the NLRP1 pathway from high levels of p62. Cytotoxicity induced by mitomycin C and mitoxantrone is stronger antagonized by p62 than by suppression of NLRP1/ASC expression. What are the molecular mechanisms underlying cytoprotection by high levels of p62 in SCC cells? We neither detected major differences between HPKs and SCC cells in autophagy nor phosphorylation of the TORC1 downstream target p70 S6 kinase. NF-κB activity is increased in SCC cells compared to HPKs. However, this is not a consequence of p62 induction as NF-κB activity is not changed in p62 knockout SCC12 cells (Fig. 5C). It has been demonstrated that Nrf2 activity is strongly increased in different types of SCC cells including those of the skin, in part by mutations in the *Nrf2* gene [37]. However, sequencing revealed only silent mutations in the genes encoding Nrf2 and Keap1 in the SCC cell lines SCC12, SCC13 and A431. Furthermore, activity of Nrf2 is only slightly increased in SCC cell lines, particularly in A431. Most importantly, knockout or overexpression of p62 did not affect Nrf2 activity in SCC12 cells or HPKs, respectively. This demonstrates that in contrast to liver cells and development of HCC, Nrf2 is not an oncogenic target of p62 in cutaneous SCCs [22, 23]. Future studies have to reveal how the p62 protein, which represents a beautiful example for complexity in molecular biology, contributes to cytoprotection in cutaneous SCCs at the molecular level independently of the NLRP1 pathway.

Our results establish p62 as a novel driver in development of SCC of the skin. This is partially mediated by suppression of the NLRP1 inflammasome pathway in established SCC cells. Due to the important role of anti-tumor immunity in controlling development of cutaneous SCCs [3], it is tempting to speculate that SCC cells in vivo might profit from attenuation of anti-tumor immune responses downstream of NLRP1 inflammasome suppression. However, this is difficult to prove in vivo, since the NLRP1 inflammasome seems to represent a young evolutionary pathway in human skin, which is not conserved in mice.

## Materials and methods

### Culture and treatment of primary cells and SCC cell lines

Isolation and culture of human primary keratinocytes (HPKs) was performed as described previously [45]. HPKs and SCC12 were grown in serum-free keratinocyte medium (KSFM, Thermo Fisher Scientific, Waltham, US-MA) supplemented with epidermal growth factor (EGF) and bovine pituitary extract (BPE). SCC13 and A431 were cultured in Dulbecco’s Modified Eagle’s Medium (DMEM, Thermo Fisher Scientific) supplemented with 10% fetal bovine serum FBS (PAN-Biotech, Aidenbach, Germany). Cells were harvested in trypsin/EDTA solution (0.05%/0.02% w/v) (Thermo Fisher Scientific) and cultured for at least 48 h before experiments. All cells were incubated at 37 °C in 5% CO_2_ and 95% humidity. HPKs and SCCs were stimulated with 0.0875 J/cm^2^ of UVB (UV802L; Waldmann, Villingen-Schwenningen, Germany), 12 μΜ mitoxantrone (Sigma-Aldrich, St. Louis, US-MO), 10 µg/ml mitomycin C (Santa Cruz, Santa Cruz, USA-CA), NF-κB inhibitor BMS-345541 (10 µM). For siRNA-mediated knockdown, cells were transfected with 10 nM siRNAs (Sigma-Aldrich, Supplementary Table 1) using Interferin (Polyplus, Illkirch, France) as a transfection reagent.

All cell lines were authenticated (Microsynth, Balgach, Switzerland) and were verified to be mycoplasma negative.

### Human biopsies

Isolation of HPKs from skin biopsy samples was performed as described previously [45, 51].

The skin biopsies were collected with informed written consent upon approval from local ethical committees (Kantonale Ethikkommission Zürich, approval numbers: 2015-0198 and 2017-00688) and were conducted according to the Declaration of Helsinki Principles.

### Manipulation of cells, stable inducible overexpression in HPK

HPKs were isolated freshly from foreskin biopsies [51] and stably genetically modified cells were generated by lentiviral transduction with constructs encoding for the gene of interest (pLenti CMV Puro DEST (w118-1); Addgene, #17452). Lentiviruses were produced in HEK 293 T cells as described previously [45, 51] using TransIT-X2 (Mirus Bio LLC, USA) transfection system. Overexpression of GFP (Addgene #17448) was used as a control. After transduction, cells were selected with 5 µg/ml puromycin (Sigma-Aldrich). Transduction and selection of HPKs was performed in coculture with antibiotic-resistant 3T3-J2 feeder cells as already described [45, 51].

### Generation of CRISPR/dCas9-KRAB-targeted SCCs

Single-stranded DNA oligonucleotides were designed on the Benchling platform (https://benchling.com). sgRNAs targeting the promoter region (Supplementary Table 2, Microsynth) were cloned into the pLV hU6-sgRNA hUbC-dCas9-KRAB-T2a-Puro (#71236, Addgene). Specific plasmids were co-transfected into HEK 293 T cells with the envelope and packaging plasmids psPAX2 (#12260, Addgene) and pMD2.G (#12259, Addgene). After 48 hours, lentiviruses were harvested and concentrated by centrifugation (16,000 *g*, 4 hours, 4 °C). The SCC12 cell line was transduced with virus resuspended in KSFM containing 2.5 μg/ml polybrene (hexadimethrine bromide) (Sigma-Aldrich). Transduction with non-targeting empty vector served as a control. Medium was changed 24 hours after transduction to KSFM medium containing supplements. After transduction, cells were selected with 5 µg/ml puromycin (Sigma-Aldrich) and expanded. Efficiency was assessed at the protein level by western blot.

### Real-time PCR

Levels of mRNA were determined by quantitative real-time PCR using the LightCycler 480 instrument and FastStart Essential DNA Green Master (Roche, Rotkreuz Switzerland) and specific primers (Microsynth, Supplementary Table 3). mRNA levels were normalized to HPRT.

### Immunoblotting

Cell culture lysates were harvested with SDS loading buffer and supernatants were precipitated with 2.5 volumes of acetone (100% w/v, Sigma-Aldrich) by overnight incubation at -20˚C followed by centrifugation for ca. 2 h (4000 *g* at 4 ˚C) and resuspended in SDS loading buffer and subjected to sonification. Proteins were separated by SDS-PAGE and analyzed by immunoblotting as previously described. The primary and secondary antibodies used are specified in Supplementary Table 4.

Full and uncropped western blots are included in the supplementary part.

### Immunohistochemistry

Specific staining for p62 on paraffin sections was performed on tissue specimens cut in 5 μm–thick sections. BOND RXm–Fully Automated Advanced Staining was used with Bond Polymer Refine Red Detection Kit (Leica Biosystems, Wetzlar, Germany), Antigen Retrieval: Leica Bond ER2 (30 min, 95 °C). Deparaffinization was done with Bond Dewax Solution (standard protocol). The slides were scanned with Aperio ScanScope (Leica Biosystems). Staining intensity scoring was performed in a blinded manner by two uninvolved scientists.

### Immunofluorescence

SCCs were seeded on round glass coverslips in 12-well plates. Cells were treated overnight with 100 µM Chloroquine (CQ) (Sigma-Aldrich) to inhibit fusion of autophagosomes and lysosomes, and visualize inhibition of autophagy. Next, cells were fixed for 30 minutes in 3% paraformaldehyde (Sigma-Aldrich) /2% sucrose (Sigma-Aldrich) solution, permeabilized for 2 minutes with 0.2% Triton X-100 in phosphate buffered saline, and blocked for 1 hour in 1% bovine serum albumin (BSA Fraction V, GE Healthcare, Chicago, US-IL) in 0.05% Tween 20 in PBS and incubated with the primary antibodies (Supplementary Table 4) for 1 hour at room temperature. Alexa Fluor® 647 conjugated secondary antibody (A21246, Thermo Fisher Scientific) was diluted (1:200) with DAPI (Sigma-Aldrich) in blocking buffer and applied for 1 hour at room temperature. Coverslips were mounted on glass slides using ProLong Gold antifade mountant (Thermo Fisher Scientific). Cells were imaged by fluorescence microscopy (Zeiss Axiocam 503 mono, Germany).

### Analysis of cell death

Cell lysis was measured by release of lactate dehydrogenase (LDH) to the supernatant by using the CytoTox 96 nonradioactive cytotoxicity assay (Promega, Madison, US-WC) according to manufacturer’s instruction, represented by percentage release of LDH (LDH in the supernatant/total LDH × 100).

### ELISA

Release of human IL-1β to the supernatant was measured by ELISA (R&D Systems, Minneapolis, US-MN) according to manufacturer’s instruction. Corresponding cell lysates were lysed for 30 min in 10% Triton X-100.

### NF-κB activity assay

NF-κB activity was measured using the TransAM NF-κB Family Kit (Active Motif, Carlsbad, US-CA) from the nuclear extract of the cells according to manufacturer’s instructions. Nuclear extracts were isolated with the Nuclear Extract Kit (Active Motif).

### Statistical analysis

All data were statistically analyzed using GraphPad Prism 9 (GraphPad Software, La Jolla, US-CA). For comparisons of two groups, two-tailed unpaired/ paired *t*-test or nonparametric Mann-Whitney test was used. Comparisons of three or more groups were performed using one-way analysis of variance with Dunnett’s post-hoc test. Differences were considered significant when P values were below 0.05 (****P < 0.001, ***P ≤ 0.001, **P ≤ 0.01, and *P ≤ 0.05, ns = not significant). All data are displayed as mean ± standard deviation (SD).

## Supplementary information

Supplementary Information

Figure S1

Figure S2

Figure S3

Figure S4

Original Data File

checklist

## Data Availability

All datasets generated and analysed during this study are included in this published article and its Supplementary Information files. Additional data are available from the corresponding author on reasonable request.
