# Supplementary material for: High p62 expression suppresses the NLRP1 inflammasome and increases stress resistance in cutaneous SCC cells
Source: Cell Death Dis. 2022 Dec 29;13(12):1077. doi: 10.1038/s41419-022-05530-0 (PMC9800582; doi:10.1038/s41419-022-05530-0)
Supplement: Supplementary file 1 — Supplementary Information [file 41419_2022_5530_MOESM1_ESM.docx]

**Supplemental Figures**


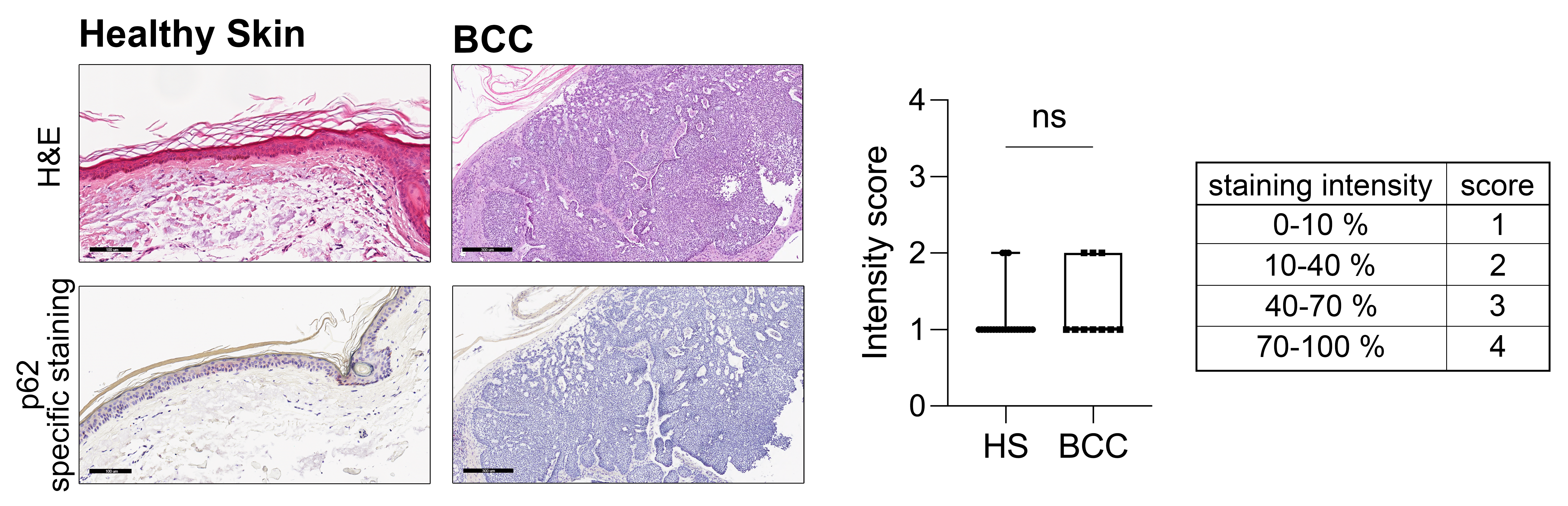


**Fig. S1 p62 expression is not increased in BCCs.**

H&E staining and p62 expression in healthy human skin (N=22) and in patient-derived BCCs (N=10) determined by immunohistochemistry. A representative staining is shown. Expression of p62 was quantified and summarized by a blinded scoring system. P values were calculated with Mann-Whitney test. (ns = not significant). Black scale bar = 100 µm (healthy skin), 300 µm (BCC). BCC, basal cell carcinoma; HS, healthy skin.


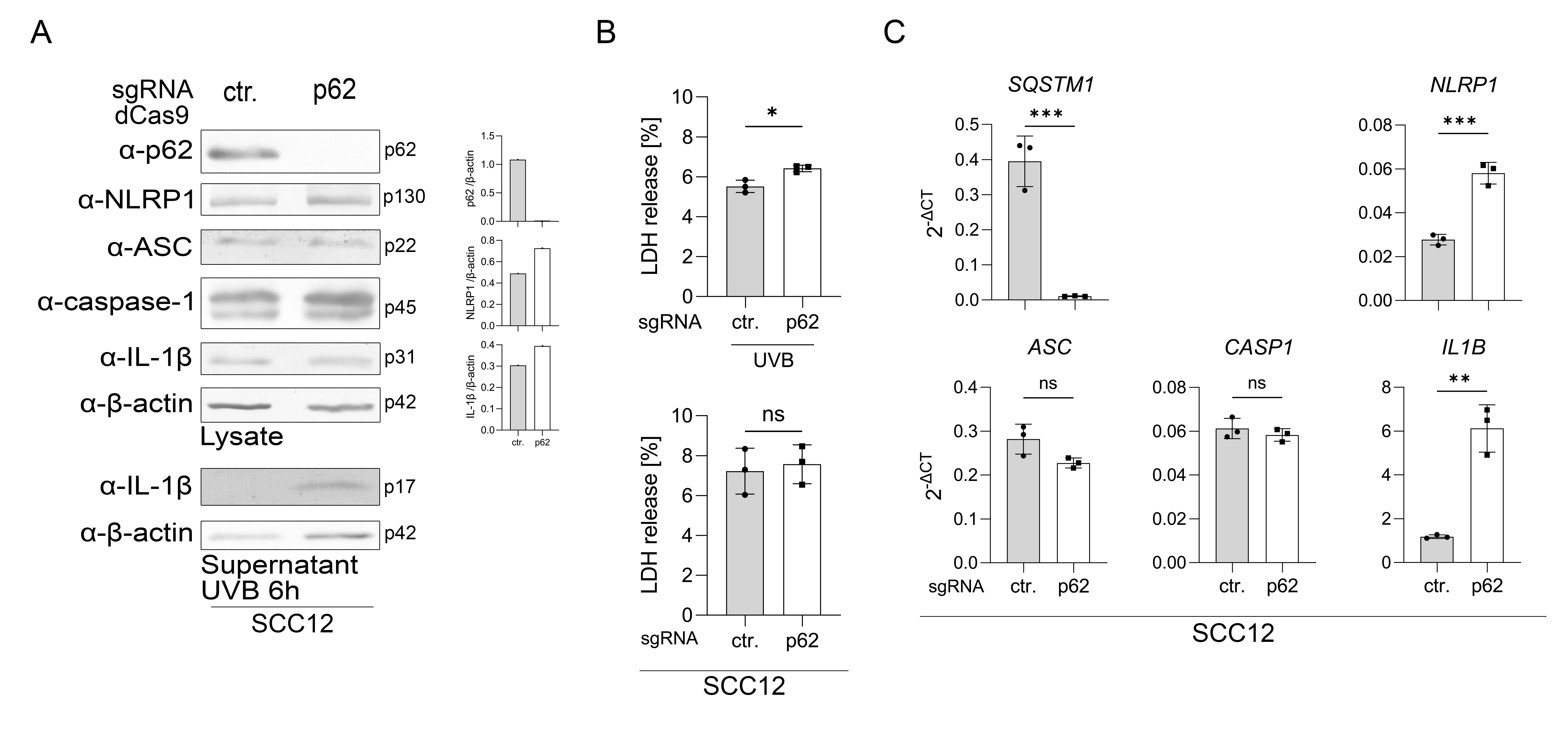


**Fig. S2 Knockout of p62 expression rescues inflammasome activation in SCC12 cells.**

(**A-C**) SCC12 cells were transfected with an empty vector (ctr.) or the RNA targeting the p62 promoter region (using a dCas9/KRAB promoter repression approach). (**A**) Western blots with lysate of mock-treated and supernatant of UVB irradiated SCC12 cells after 6 h. (**B**) LDH released 6 h after UVB irradiation (upper panel) or after mock treatment (lower panel). (**C**) mRNA expression of the indicated genes determined by qPCR in mock-treated cells. Data are represented as mean ± SD of three (**B, C**) experiments, or are representative of three (**A**) independent experiments. P values were calculated with two-tailed paired (N=3) (**B, C**) *t* test. (∗∗∗P ≤ 0.001, ∗∗P ≤ 0.01, and ∗P ≤ 0.05, ns = not significant).


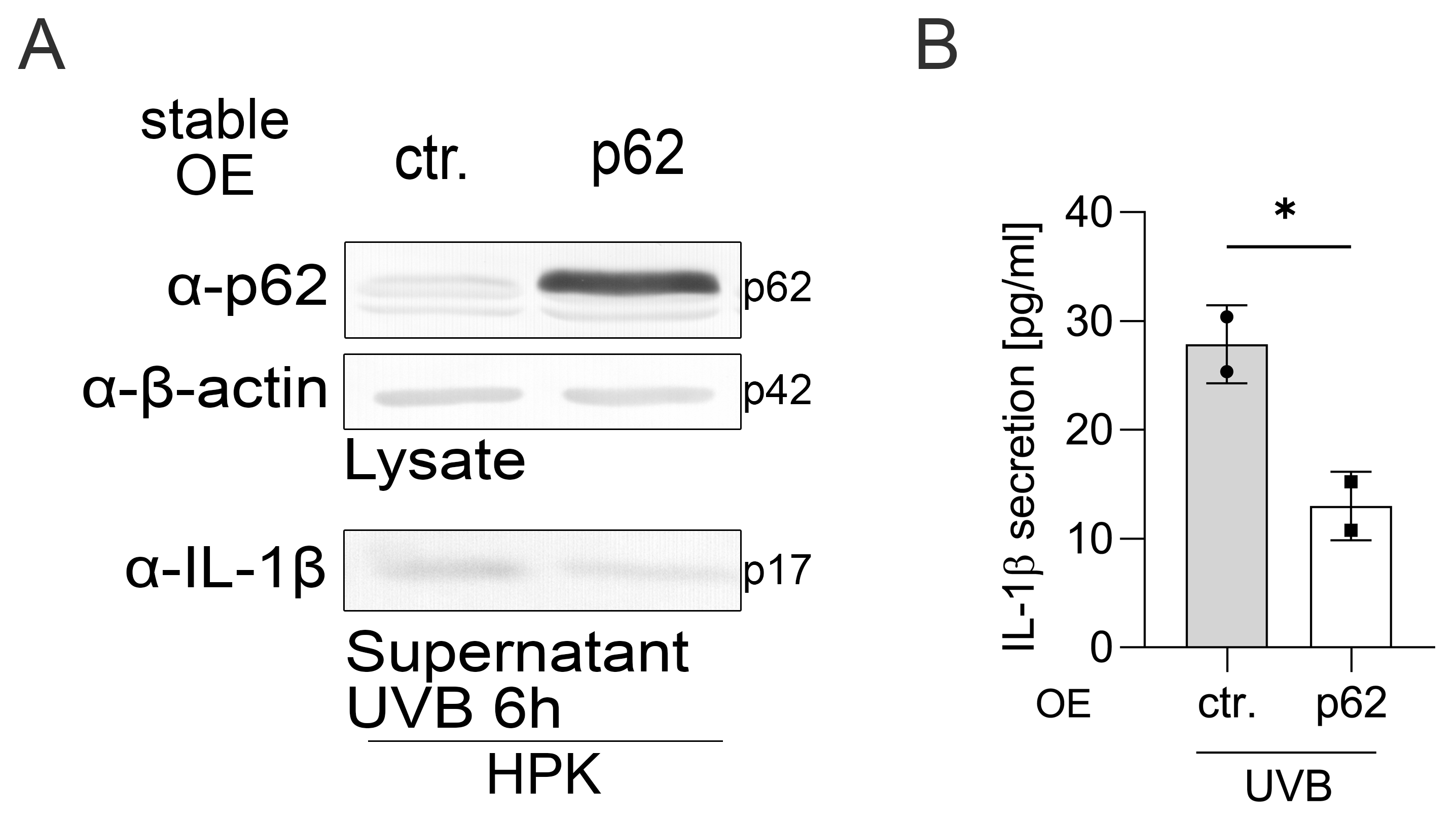


**Fig. S3 Overexpression of p62 dampens UVB-induced NLRP1 activation in HPKs.**

(**A-B**) HPKs were transduced with a lentivirus encoding eGFP (ctr.) or p62. (**A**) Western blots showing protein expression of the indicated genes using lysates of mock-treated cells or supernatant 6 h after UVB irradiation. (**B**) ELISA for quantification of IL-1β secreted 6 h after UVB irradiation. Data are represented as mean ± SD of two (**B**) experiments, or are representative of two (**A**) independent experiments. P values were calculated with two-tailed paired (N=2) (**B**) *t* test. (∗P ≤ 0.05).

**Fig. S4 Autophagy, mTORC1 and Nrf2 are not deregulated in SCC cell lines.**

(**A and B**) HPKs from three different donors and SCC cell lines SCC12, SCC13 and A431 were mock-treated or with the autophagy inhibitor chloroquine overnight. (**A**) Western blot for expression of the indicated proteins. (**B**) Immunofluorescence for LC3B puncta accumulation (red), co-stained with DAPI (blue). (**C**) Western blot for expression of the indicated proteins in HPKs and SCC cell lines. (**D**) Sanger sequencing of the *Nrf2* and *Keap1* gene in SCC12, SCC13 and A431. Shown are only mutations. Data are representative of three (**A**) or two (**B,** **C**) independent experiments.

**Materials and Methods - Supplemental Tables**

| **siRNA** | **oligo name** | **sequence 5'-3'** |
| --- | --- | --- |
| Ctr.-1 | Mission siRNA Universal negative control #1 (SIC001) |  |
| Ctr.-2 | Casp-5 3823180 | UGGAUAACUUCGUGAUAAA [dTdT] |
| p62-1 | SASI_Hs01_00118616 | CGUCUACAGGUGAACUCCA [dTdT] |
| p62-2 | SASI_Hs01_00118618 | GGCUGAAGGAAGCUGCCUU [dTdT] |

**Supplementary Table 1.** List of siRNA sequences used for knock down experiments.

| **sgRNA targeting promoter** | **sequence 5'-3'** |
| --- | --- |
| p62 | Forward CACCGTGAAGGGGCCTCTGCAGGG  Reverse AAACCCCTGCAGAGGCCCCTTCAC |

**Supplementary Table 2.** The sequence of sgRNAs targeting the promoter region of p62 used for generation of CRISPR/dCas9-KRAB-targeted SCCs.

| **Real-time PCR primers** | **sequence 5'-3'** |
| --- | --- |
| *ASC (PYCARD)* | Forward CGCGAGGGTCACAAACGT  Reverse TGCTCATCCGTCAGGACCTT |
| *CASP1* | Forward TCCCTAGAAGAAGCTCAAAGGATATG  Reverse CGTGTGCGGCTTGACTTG |
| *HMOX1* | Forward ACTGCGTTCCTGCTCAACATC  Reverse GCTCTGGTCCTTGGTGTCATG |
| *HPRT* | Forward ATTGTAATGACCAGTCAACAGGG  Reverse GCATTGTTTTGCCAGTGTCAA |
| *KEAP1* | Forward CTGGAGGATCATACCAAGCAGG  Reverse GAACATGGCCTTGAAGACAGG |
| *IL1B* | Forward CACGATGCACCTGTACGATCA  Reverse GTTGCTCCATATCCTGTCCCT |
| *NLRP1* | Forward CAGGCAGCACAGATCAACAT  Reverse GTGACCTTGAGGACGGAGAA |
| *NQO1* | Forward GTGATATTCCAGTTCCCCCTGC  Reverse AAGCACTGCCTTCTTACTCCGG |
| *NFE2L2/NRF2* | Forward CCAGGTTGCCCACATTC  Reverse TCCCAAACTTGGTCAATGTCC |
| *SQSTM1/p62* | Forward GACTACGACTTGTGTAGCGTC  Reverse AGTGTCCGTGTTTCACCTTCC |

**Supplementary Table 3.** List of primers used for quantitative real time PCR.

| **Antibody** | **Order number** | **Application** |
| --- | --- | --- |
| p62 | sc-28359 (Santa Cruz) | WB |
| Nrf2 | ab62352 (Abcam, Cambridge, United Kingdom) | WB |
| Keap1 | sc-15246 (Santa Cruz) | WB |
| Nqo1 | ab28947 (Abcam) | WB |
| NLRP1 | 679802 (BioLegend, San Diego, US-CA) | WB |
| ASC | ALX-210-905-C100 (Enzo Life Sciences, Farmingdale, US-NY) | WB |
| Caspase-1  Caspase-1 (p20) | sc-622 (Santa Cruz)  AG-20B-0048 (Adipogen) | WB |
| IL-1β | MAB201 (R&D) | WB |
| IL-18 | PM014 (MBL, Woburn, US-MA) | WB |
| GSDMD | NBP2-33422 (Novus, Littleton, US-CO) | WB |
| LC3B | ab51520 (Abcam) | WB, IF |
| NF-κB p65 | sc-372 (Santa Cruz) | WB |
| Phospho-NF-κB p65 (Ser536) | 3033 (Cell Signaling, Danvers, US-MA) | WB |
| β-actin | A5441 (Sigma) | WB |
| Phospho-p70 (Thr389) | 9234 (Cell Signaling) | WB |
| Phospoho-AKT (Ser473) | 4060 (Cell Signaling) | WB |
| p62 | ab207305 (Abcam) | IHC |
| Isotype Control | ab172730 (Abcam) | IHC |
| Anti-Rabbit IgG (Fc), AP Conjugate | s373b (Promega) | Secondary AB, WB |
| Anti-Mouse IgG (H+L), AP Conjugate | s372b (Promega) | Secondary AB, WB |
| Anti-Goat IgG, AP Conjugate | V115A (Promega) | Secondary AB, WB |
| Anti-Rabbit IgG (H+L) Alexa Fluor 647 | A21246 (Thermo Fisher Scientific) | Secondary AB, IF |
| Anti-Rabbit IgG human ads-BIOT | 4010-08 (Southern Biotech, Birmingham, US-AL) | IHC |

**Supplementary Table 4.** List of antibodies used for immunoblotting, immunohistochemistry, and immunofluorescence.
