# Supplementary figures and images for: High p62 expression suppresses the NLRP1 inflammasome and increases stress resistance in cutaneous SCC cells
Source: Cell Death Dis. 2022 Dec 29;13(12):1077. doi: 10.1038/s41419-022-05530-0 (PMC9800582; doi:10.1038/s41419-022-05530-0)

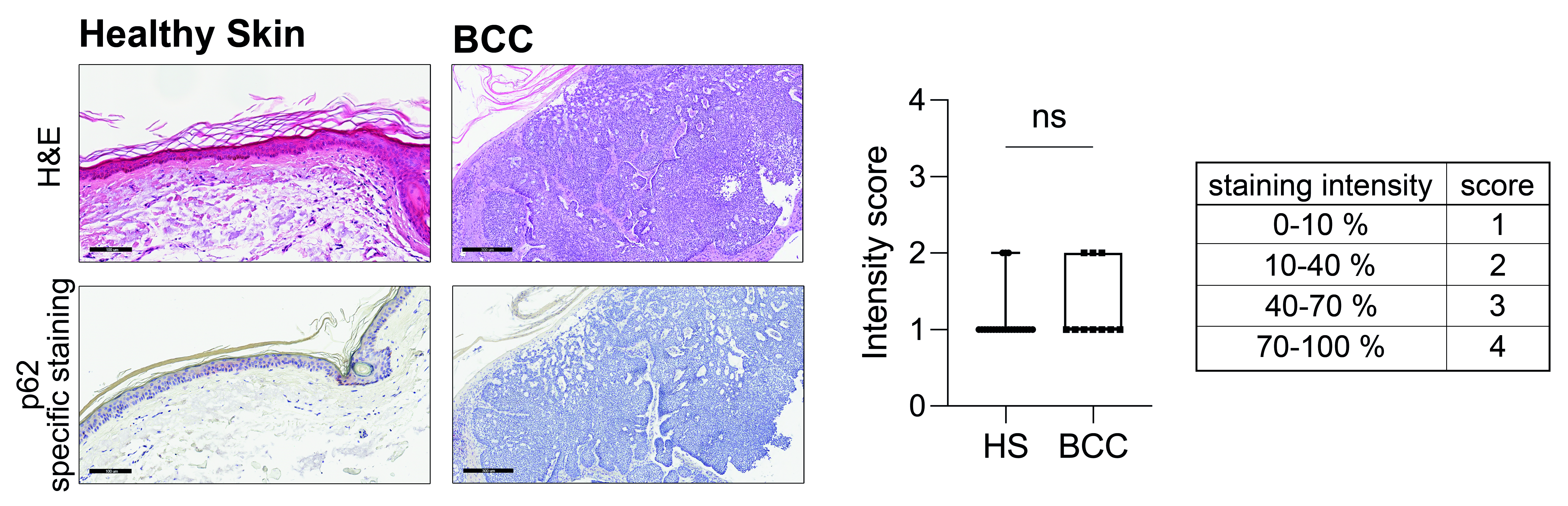

Supplement: Supplementary file 2 — Figure S1 [file 41419_2022_5530_MOESM2_ESM.tif]

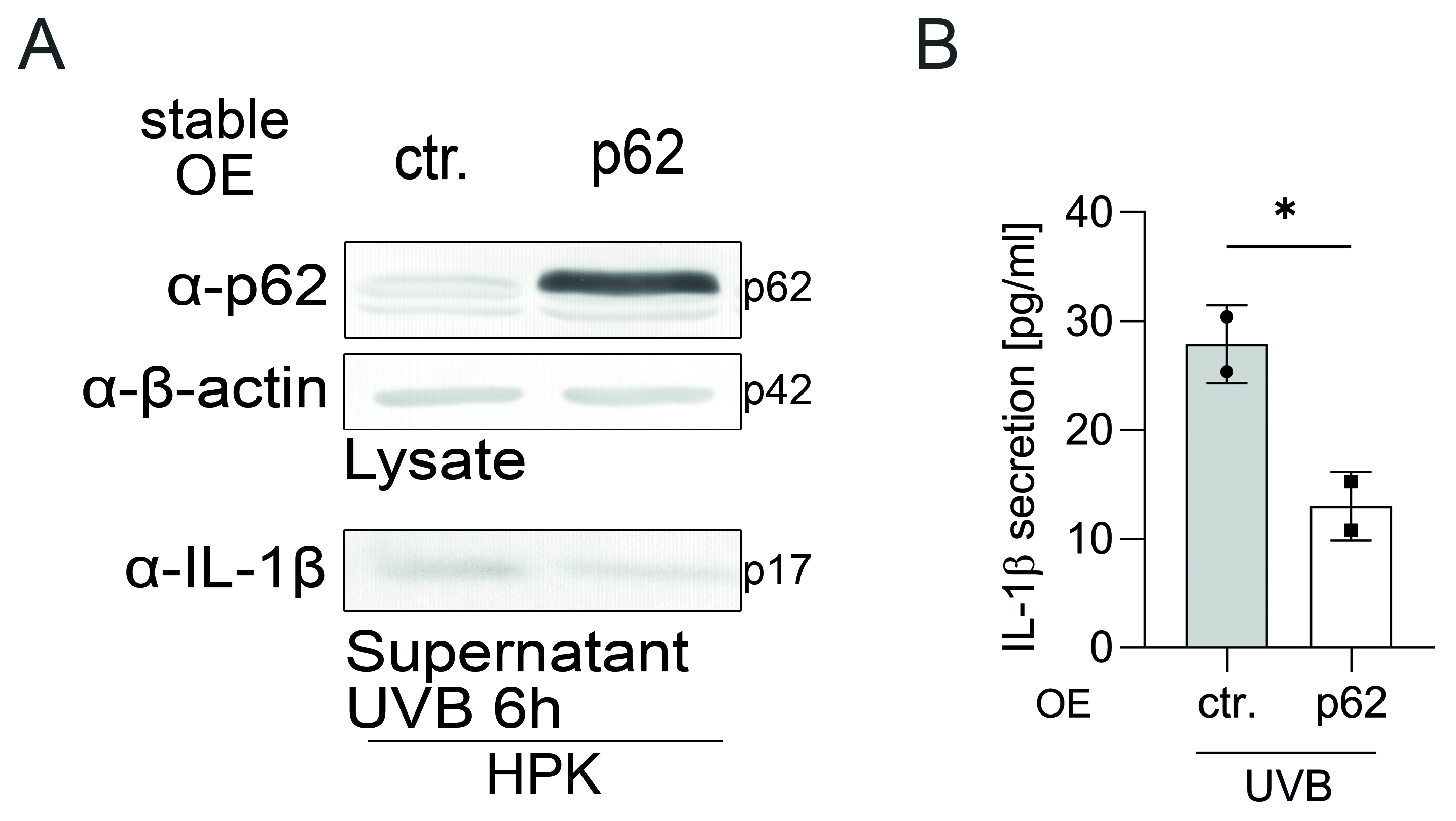

Supplement: Supplementary file 4 — Figure S3 [file 41419_2022_5530_MOESM4_ESM.tif]
