# Supplementary material for: High p62 expression suppresses the NLRP1 inflammasome and increases stress resistance in cutaneous SCC cells
Source: Cell Death Dis. 2022 Dec 29;13(12):1077. doi: 10.1038/s41419-022-05530-0 (PMC9800582; doi:10.1038/s41419-022-05530-0)

Figure 1C

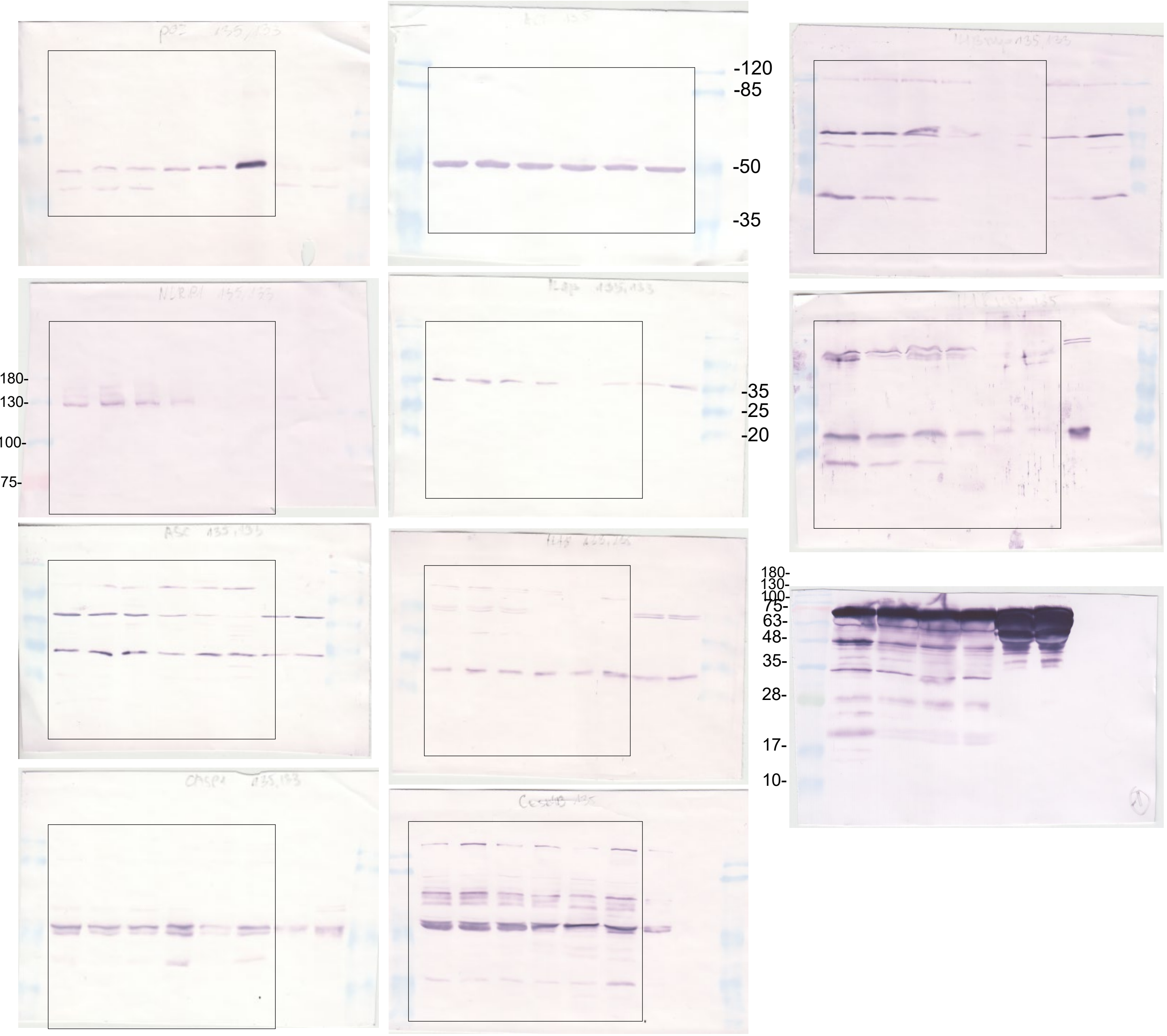

Figure 2A

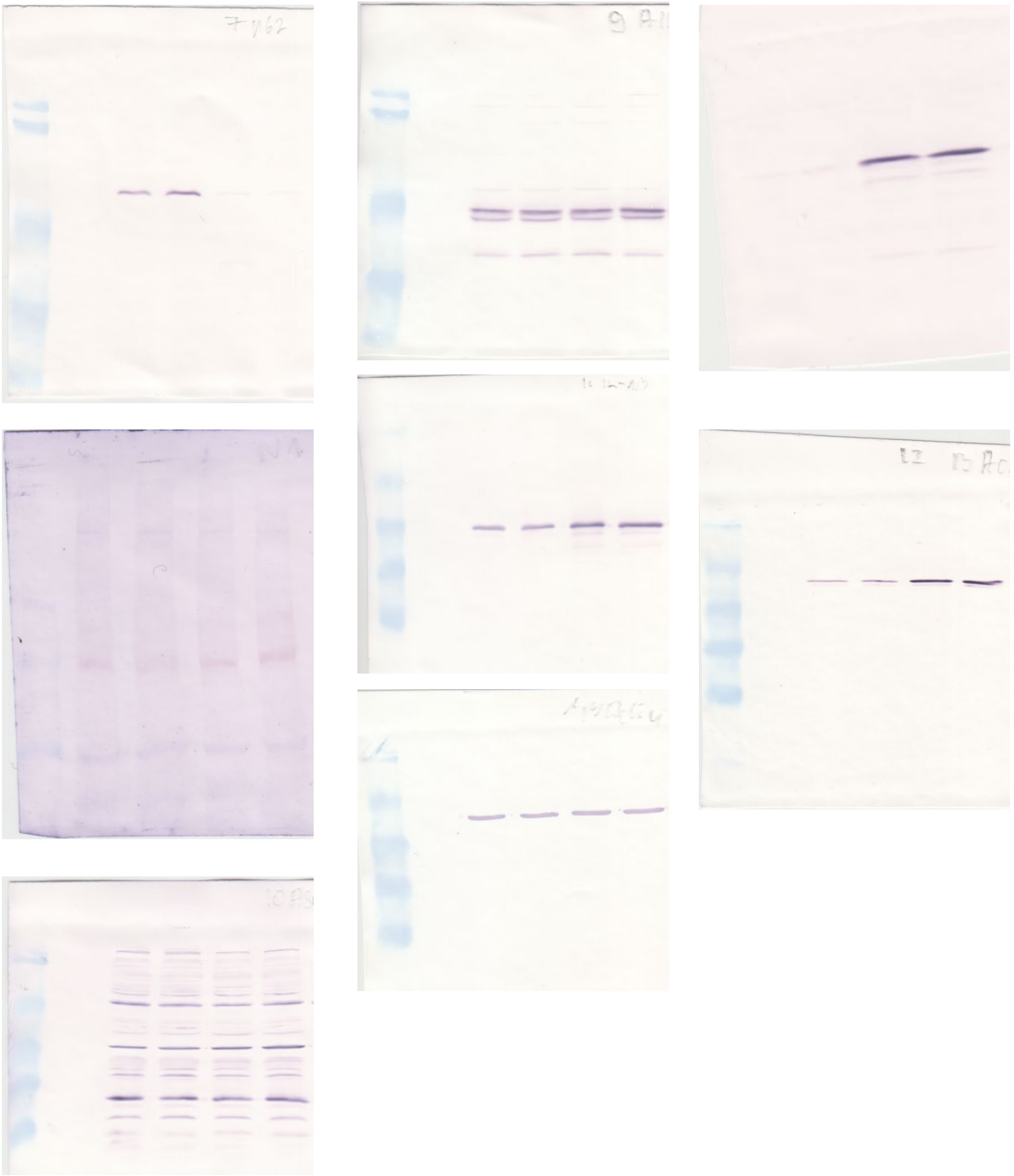

Figure 3A

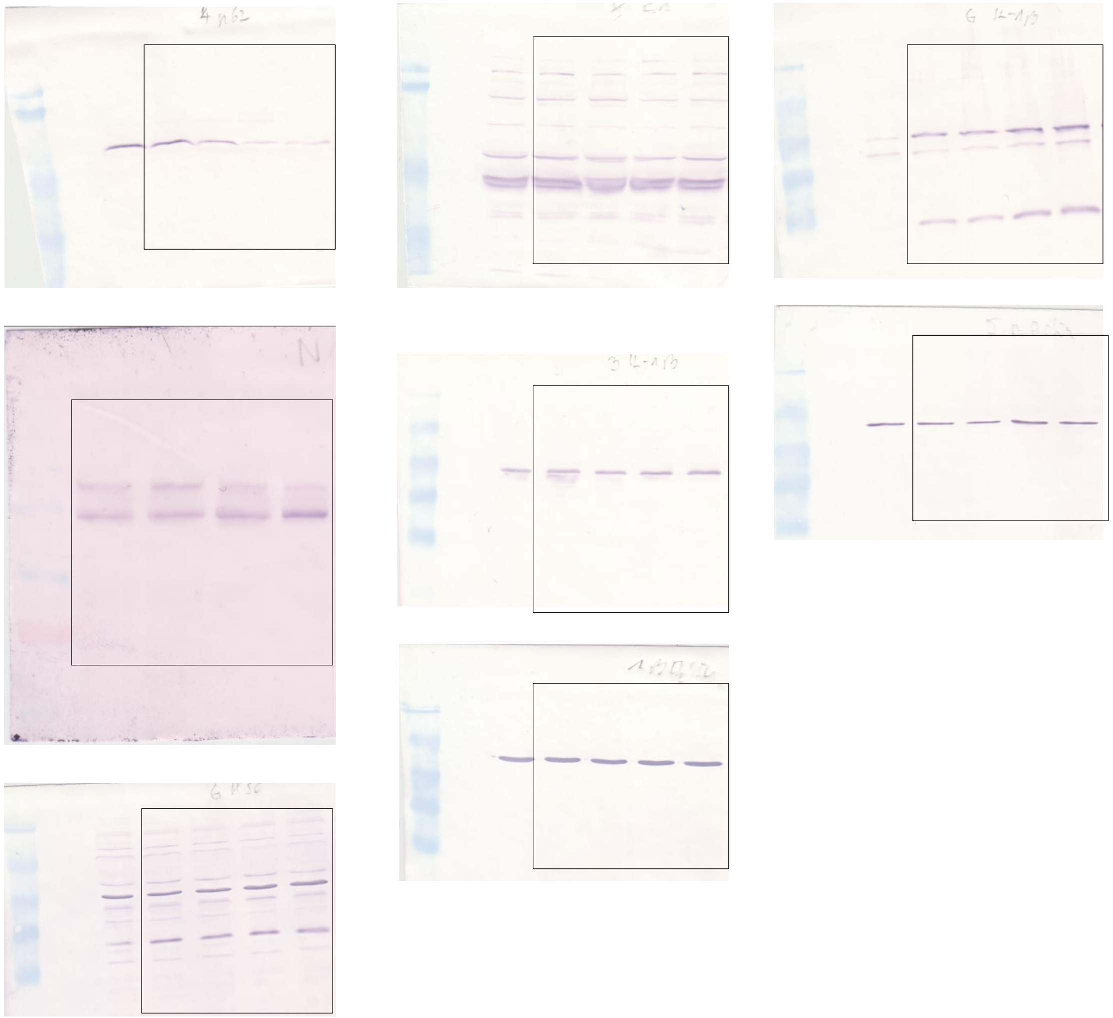

Figure 4A

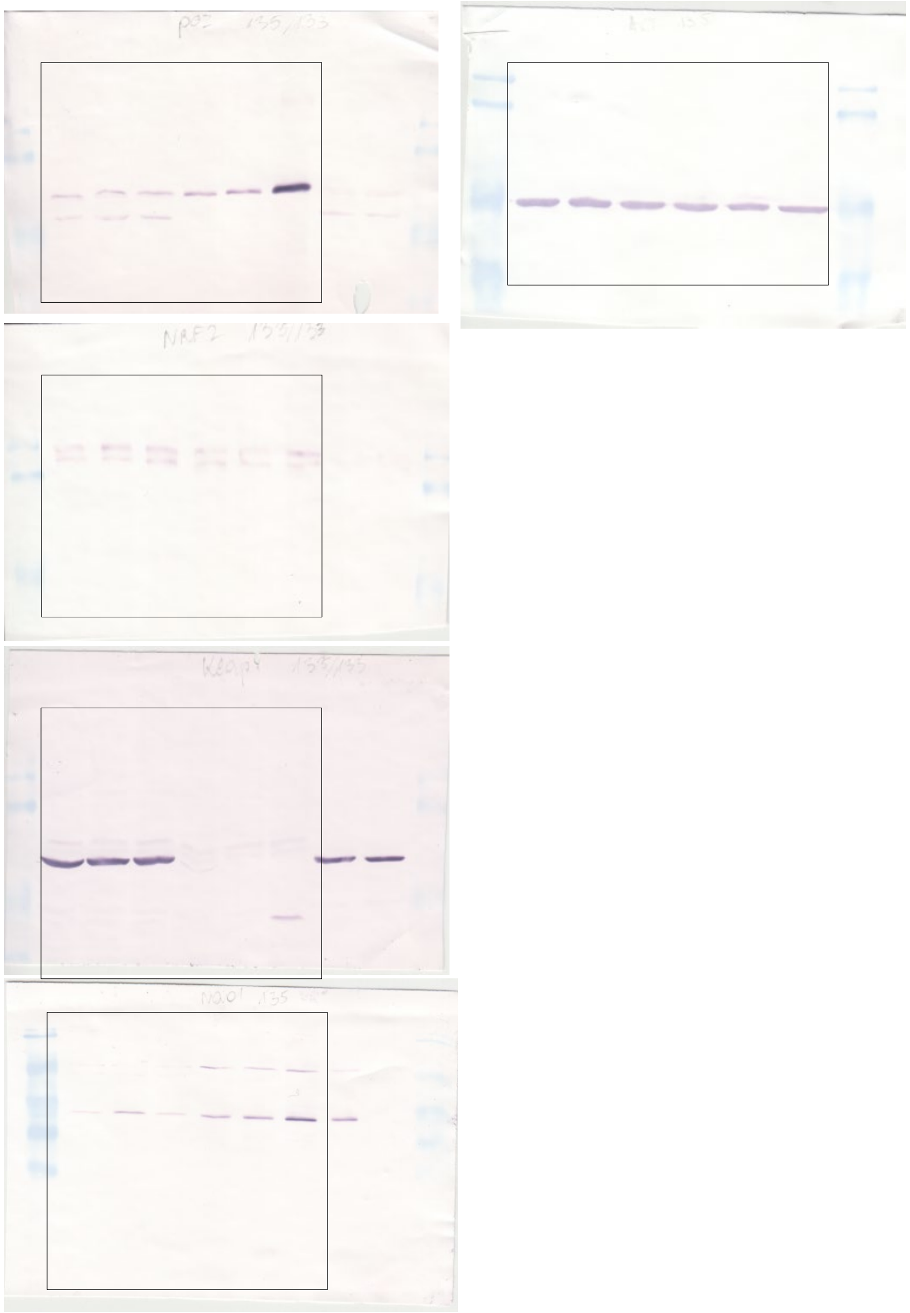

Figure 4C

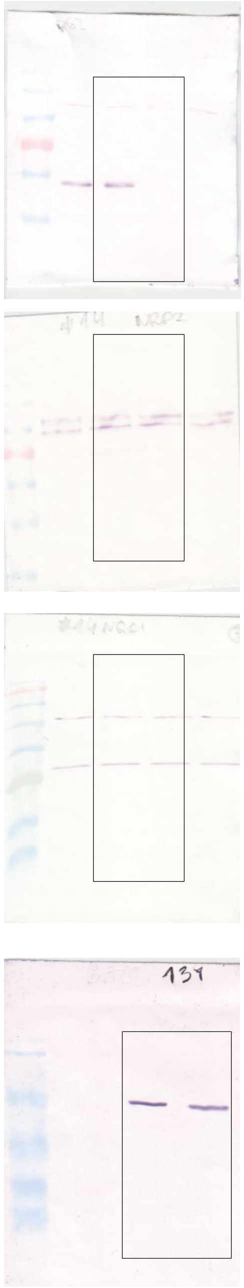

Figure 4E

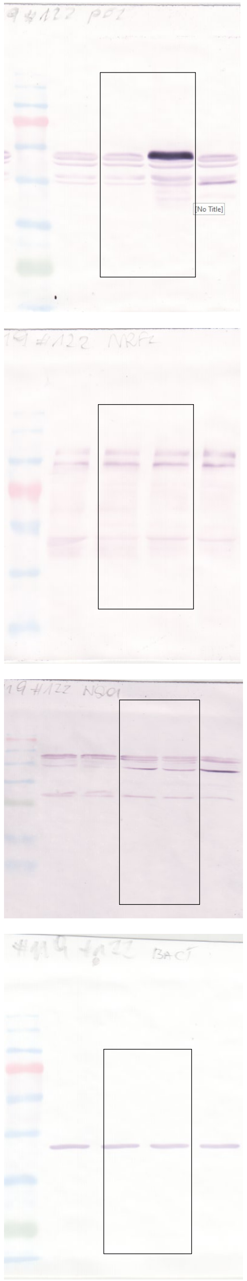

Figure 5A

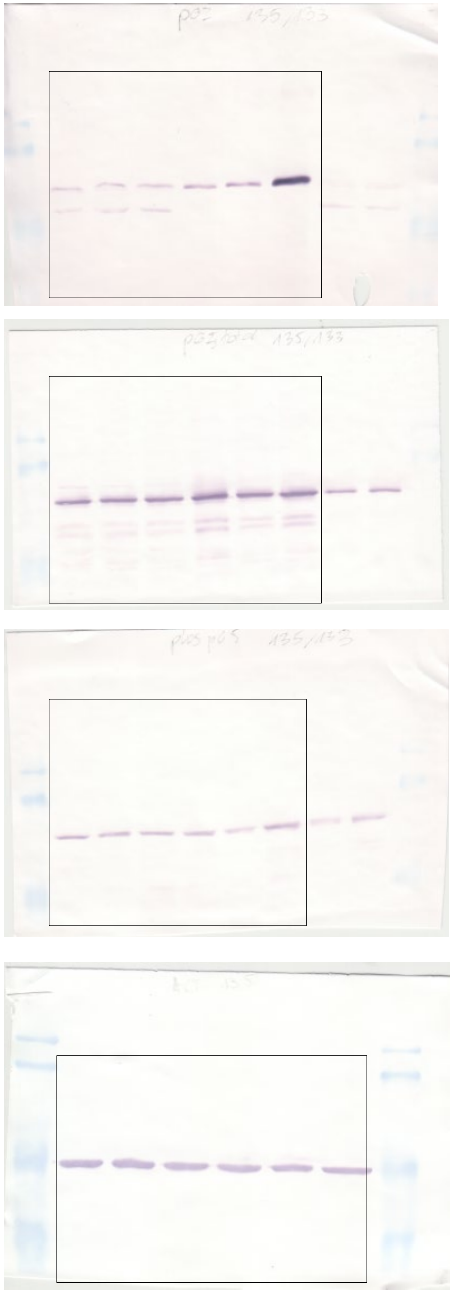

Figure 5D

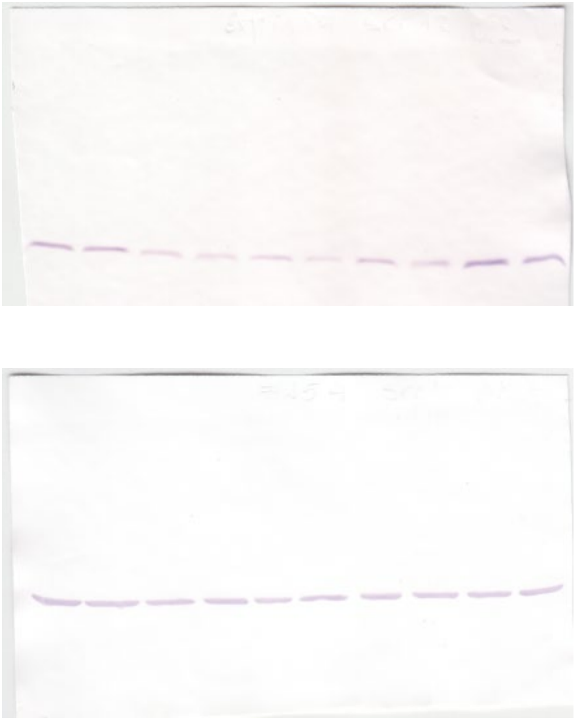

Figure 5E

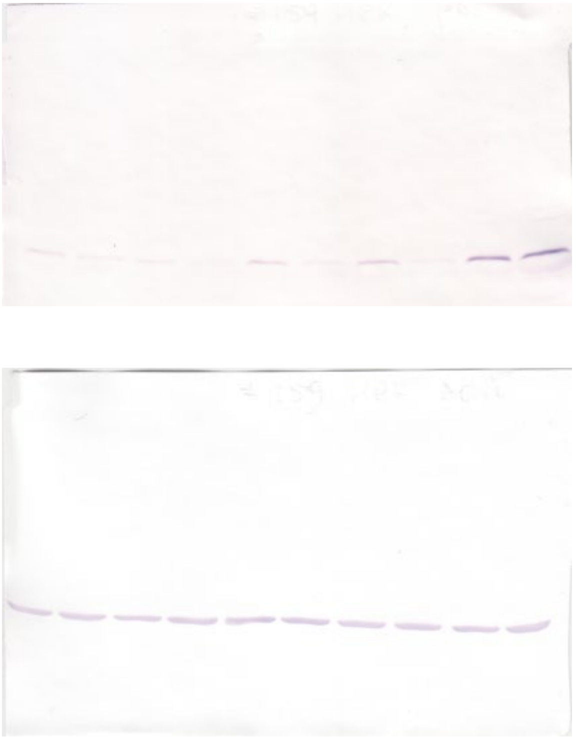

Figure 6H

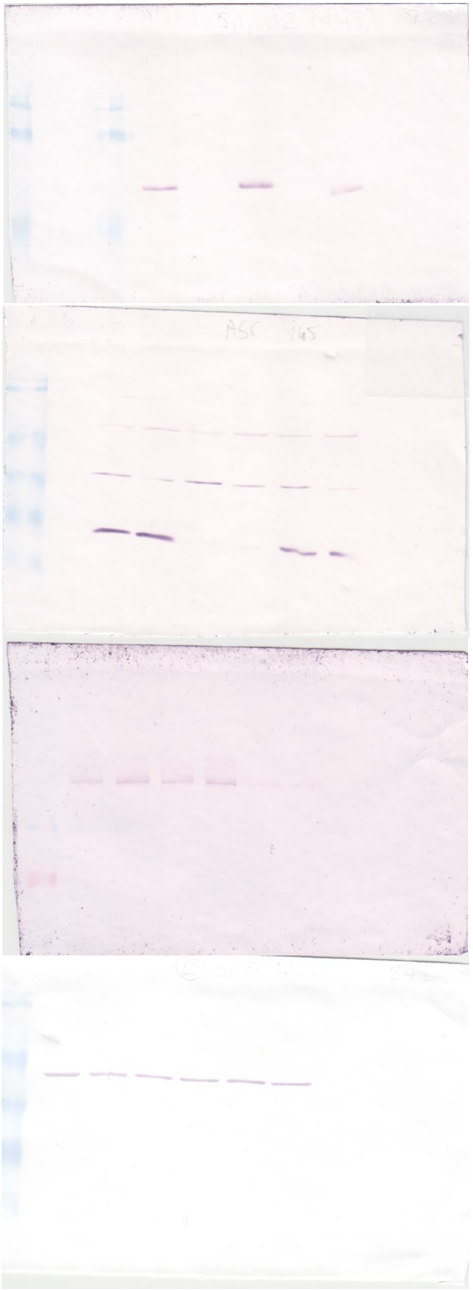

Figure S2A

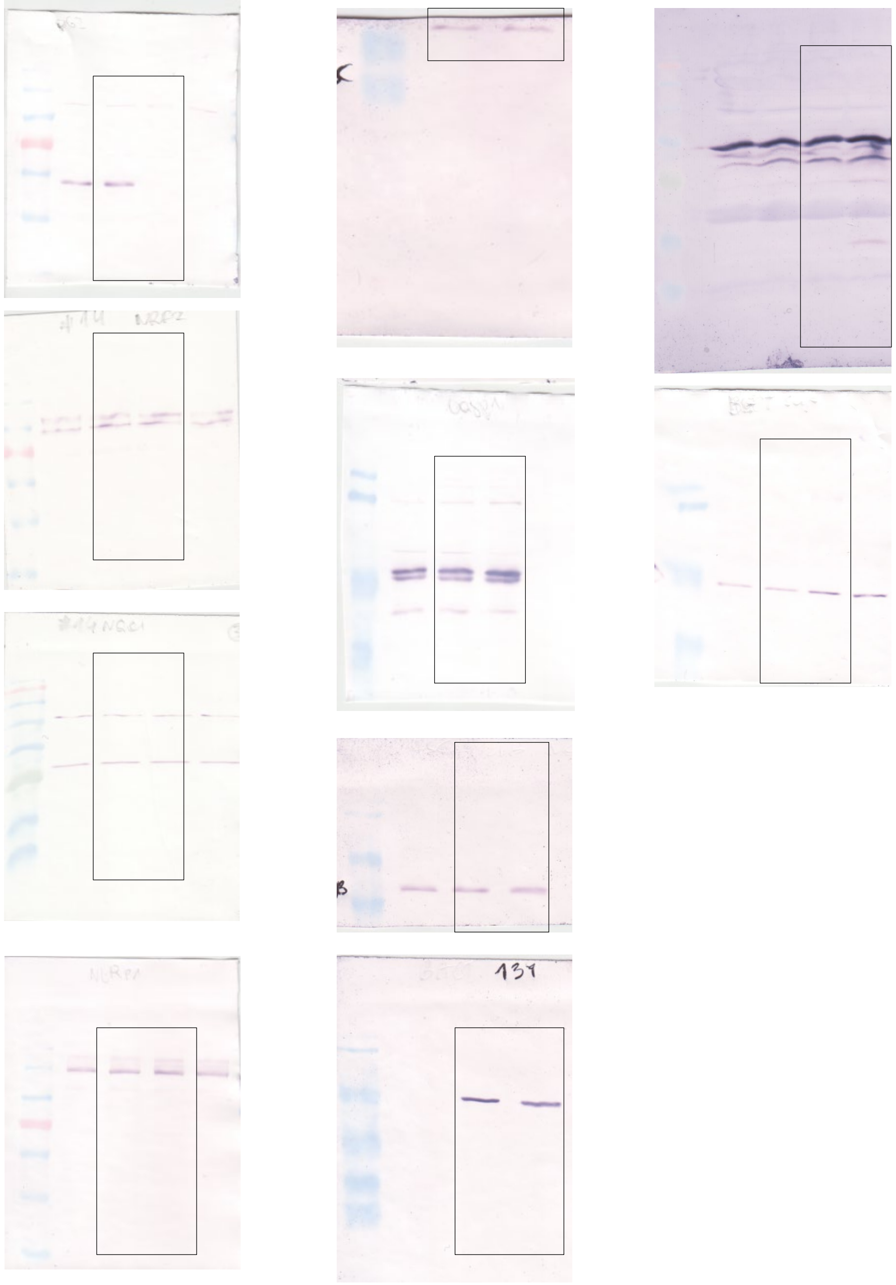

Figure S3A

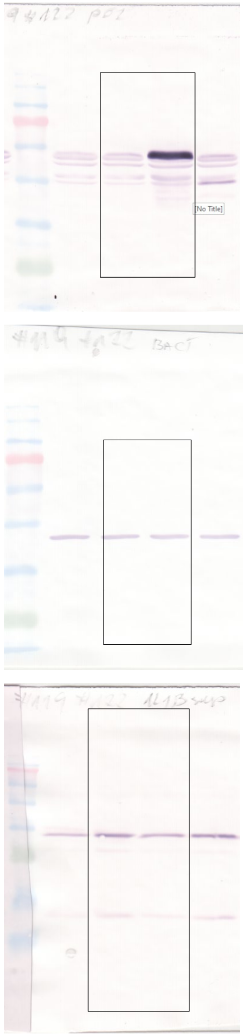

Figure S4A

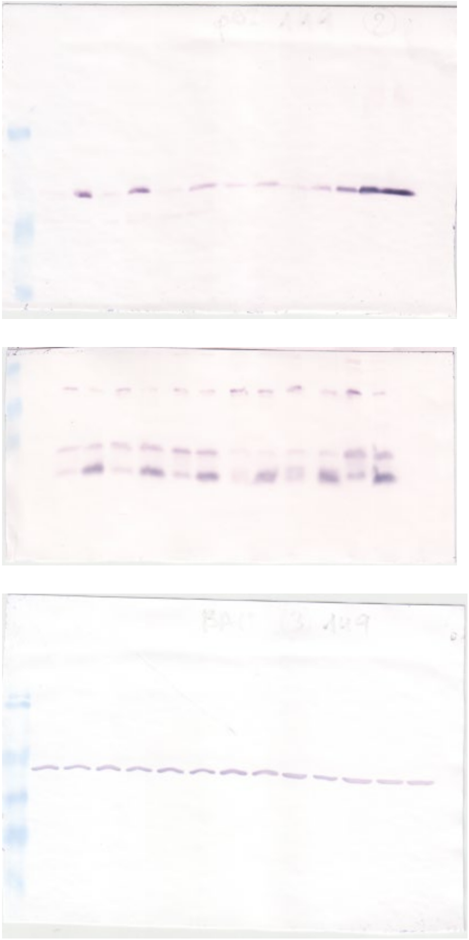

Figure S4C

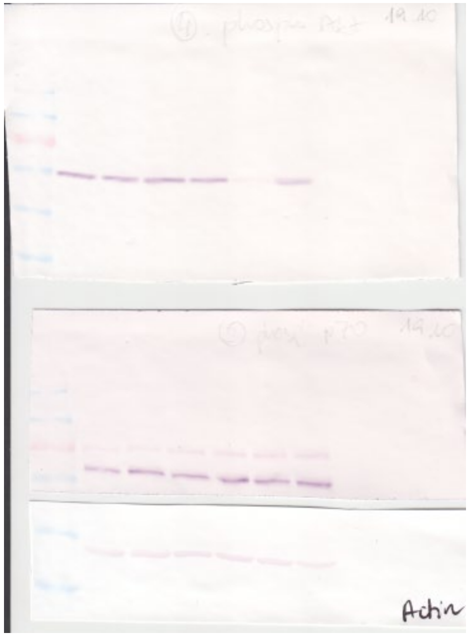

Supplement: Supplementary file 6 — Original Data File [file 41419_2022_5530_MOESM6_ESM.pdf]
